# Sociodemographic Differences in Smoking Behaviours by Migration Background: Insights From the National Swiss Health Survey

**DOI:** 10.3389/ijph.2026.1609268

**Published:** 2026-04-20

**Authors:** Kris Schürch, Tayisiya Krasnova, Lyra Egan, Tara Gückel, Lily Davidson, Lars Lenze, Annika Frahsa

**Affiliations:** 1 Graduate School for Health Sciences, University of Bern, Bern, Switzerland; 2 Swiss Association for Tobacco Control (AT Switzerland), Bern, Switzerland; 3 Institute of Social and Preventive Medicine, University of Bern, Bern, Switzerland; 4 The University of Sydney The Matilda Centre for Research in Mental Health and Substance Use, Camperdown, NSW, Australia

**Keywords:** migration and health, smoking behaviour, social inequities, Switzerland, tobacco control

## Abstract

**Objectives:**

This study aimed to examine how migration background is associated with current smoking and whether this relationship varies by sex, age, and education.

**Methods:**

We analysed data from 19,441 participants of the 2022 Swiss Health Survey, an official, national cross-sectional dataset. Current smoking (yes/no) was the outcome, and migration background (none, 1st generation, 2nd or higher generation) the primary exposure. Multivariable logistic regression models adjusted for sociodemographic and behavioural covariates, were followed by stratified analyses by sex, age, and education.

**Results:**

Compared to people without a migration background, the odds of current smoking were elevated among those with a 1st-generation background (OR = 1.42, 95% CI: 1.28–1.49) and 2nd or higher generation (OR = 1.75, 95% CI: 1.48–2.06). Stratified analyses showed that the higher odds of current smoking among people with a migration background were particularly pronounced among younger adults (15–24, 25–34 years), and among people with lower educational attainment.

**Conclusion:**

Migration background contributes to smoking inequalities in Switzerland and intersects with other social factors, such as age and education. Elevated risks among people with 2nd or higher-generation migration backgrounds in younger age groups and those with lower educational attainment demonstrate the need for culturally adapted, equity-oriented prevention strategies and stronger national tobacco control policies.

## Introduction

Across most populations, tobacco smoking follows a marked social gradient: people with lower education or socioeconomic position are more likely to smoke and less likely to quit [1–4]. While these social gradients in smoking are well established internationally, their manifestation within specific national contexts depends strongly on population composition (e.g., migration background, sex distribution, age structure) and tobacco control environments. Switzerland represents a particularly relevant context for examining these dynamics, given its persistently high smoking prevalence, large population with migration backgrounds, and comparatively weak tobacco control policies by European standards [5]. While some studies have examined socioeconomic inequalities in smoking within the Swiss context [6], the most recent national analysis by Wehrli et al. [7] was descriptive and reported higher smoking rates among individuals with lower education and among non-Swiss citizens [7]. Evidence specifically documenting how migration background intersects with smoking and other social determinants, however, remains limited. Given that 41% of Switzerland’s population has a migration background [8], and that the national tobacco control framework is comparatively weak, [5], understanding how migration background shapes the social patterning of smoking is an urgent public health priority.

Importantly, smoking is common in Switzerland and contributes considerable health and economic impacts. Cigarette smoking is the most common form of tobacco use in Switzerland, with 24% of residents aged 15 years or older reporting current smoking in 2022 [9, 10]. Nationally, smoking remains the leading preventable cause of premature mortality, accounting for about 10,000 deaths annually, which is equivalent to nearly 15% of all deaths and generates substantial economic costs estimated at up to CHF 6.1 billion per year [11–13]. Beyond these population-level burdens, tobacco smoking is associated with reduced quality of life and adverse mental health outcomes [14–16].

Despite sustained tobacco control efforts, including WHO MPOWER measures, which provide countries with guidelines to reduce tobacco demand (e.g., raising taxes), that have reduced smoking in higher socio-economic groups, equitable benefits have not been achieved across all populations [17]. People with migration backgrounds and others facing social or economic disadvantages often encounter barriers to accessing prevention and cessation services [18–20], with a few exceptions where targeted and equity-oriented approaches have been implemented [21]. In the Swiss context, these persistent differences highlight the need to better understand how social position, particularly migration background, relate to smoking behaviours.

Building on this literature, although smoking behaviours are influenced by multiple demographic determinants, including sex, and age, as well as social determinants, including education, and employment, or modifiable health risk behaviours including alcohol or other drug use [22–24], evidence also suggests that migration background may be an intersecting axis of inequality [25–27]. International studies indicate that people with migration backgrounds, especially males and those with lower education, are more likely to smoke, hold more positive attitudes towards smoking, and report lower smoking-related health literacy. These patterns are further shaped by sex, age (e.g., youth), stress, acculturation processes, or linguistic isolation [28–30], suggesting that migration background may modify established social gradients in smoking, though the direction and magnitude of this relationship potentially depend on the social and policy context in which people with migration backgrounds live. Yet this hypothesis has not been empirically tested using recent, nationally representative Swiss data. Specifically, there is a lack of up-to-date Swiss evidence examining whether the association between migration background and current smoking differs by sex, age and education.

To address this gap, this study examines migration background as the primary exposure, conceptualised as a social position within an intersectional framework [31]. In this framework, modifiable health risk behaviours such as smoking are shaped by overlapping sociodemographic determinants, including sex, age education, and migration status that interact rather than act independently [32]. We thus assess whether the association between migration background and current smoking differs by sex, age, and education, which are treated as potential effect modifiers on prior evidence, while other sociodemographic factors (marital status, residence, language region, and employment) and modifiable health risk behaviours (alcohol and drug use) are included as covariates [23, 33–37]. This focus is particularly relevant in Switzerland, a country with a large migrant population coinciding with comparatively weak tobacco control [5, 8].

### Objectives and Hypotheses

This study aims to examine the association between migration background and current smoking in Switzerland using recent, nationally representative survey data. We assess whether this association differs by sex, age, and education, and whether migration background amplifies or attenuates the social gradient in smoking.

We hypothesise that people with a migration background will have higher odds of current smoking than people without, with higher odds of current smoking among males with a migration background than females with a migration background, and among people with a migration background with lower education in comparison to those with a higher education. We further hypothesise that the association between migration background and current smoking differs by age, with stronger associations observed in younger age groups.

## Methods

### Study Design and Data Source

We used data from the 2022 Swiss Health Survey (SHS), a large, nationally representative, cross-sectional survey of residents aged ≥15 years in private households, conducted by the Federal Statistical Office [38]. The SHS applies a stratified random sampling design and survey weights to reflect the Swiss resident population. The data was collected through computer assisted telephone interviews in German, French, and Italian. Of 21,930 respondents, 19,441 were included in the current analytic sample, after excluding respondents with missing covariates related to current smoking, and other included sociodemographic factors.

### Measures

Detailed description of variable coding is provided in Supplementary Material 1.

### Outcome

Smoking status was derived from SHS questions on current tobacco use. People were asked: “Do you smoke, even if only occasionally? (including heated tobacco products such as IQOS, but excluding e-cigarettes).” Those who responded “Yes” were classified as current smokers; all others were classified as non-smokers. Smoking status was therefore analysed as a binary outcome (0 = non-smoker, 1 = current smoker).

### Exposure Variables and Effect Modifiers

The primary exposure was migration background, classified according to the Swiss Federal Statistical Office typology based on respondents’ and parents’ countries of birth and citizenship. People were grouped into three categories: no migration background (reference group), 1st generation migration background (born abroad), and 2nd or higher-generation migration background (born in Switzerland with at least one parent born abroad). Detailed coding of the Federal Statistical Office schema is shown in Supplementary Material 1, Supplementary Table S1. Based on prior evidence sex, age, and education were specified *a priori* as potential effect modifiers of the migration background–current smoking association. Additional sociodemographic variables included marital status, residential setting (urban, peri-urban, rural), language region (German, French, Italian), employment status. Modifiable health risk behaviours included alcohol use, and other drug use (see Supplementary Material 1, Supplementary Table S2).

### Statistical Analyses

The SHS data were analysed with the software RStudio (Version 2025.05.1 + 513) [39]. In a first step, data were cleaned and missing data were handled with complete-case analysis. Differences in distributions between included and excluded cases were descriptively assessed using Mann-Whitney-U (ordinal variables) and chi-squared tests (nominal variables) (see Supplementary Material 2, Supplementary Table S1). In a second step, associations between migration background (none, 1st generation, 2nd generation) and current smoking were examined using a multivariable logistic regression model adjusting for all included covariates. We additionally conducted an adjusted analysis, comparing 1st generation to 2nd or higher migration background. The overview in associations between current smoking status and sociodemographic factors, alcohol and drug use can be viewed in Supplementary Material 3. In a third step, before conducting stratified analyses, effect modification by sex, age group, and education was formally assessed by including interaction terms between migration background and each potential modifier in multivariable logistic regression models using survey weighted Wald tests. When stratifying by one variable, the models were adjusted for the other two and for included sociodemographic (migration background, marital status, employment status, residence area, and language region) and modifiable health risk behaviour covariates (alcohol and drug use). For instance, models stratified by age were adjusted for sex and education, as well as migration background, marital status, employment status, residence area, language region, alcohol and drug use. Supplementary Material 4 provides the full RStudio code book used for the Mann-Whitney-U,chi-squared and Wald tests, as well as the subsequent statistical models.

## Results

### Characteristics of the Study Population

Of the 21,930 individuals in the original dataset, 2,489 (11.3%) were excluded due to missing covariate data, leaving 19,441 people for analysis. Formal statistical comparisons between included and excluded participants indicated systematic differences across several characteristics (see Supplementary Material 2, Supplementary Table S1).

Just over half of the people in the total study sample were female (53.7%) (see Table 1). The largest age groups were 45–54 (19.0%) and 55–64 (20.7%). Most were married or in a registered partnership (55.8%). Two-thirds in the sample had no migration background (66.3%), followed by 1st generation (26.2%), and 2nd or higher generation (7.4%). Regarding education, 41.2% held a tertiary degree, 45.6% secondary, and 13.2% compulsory schooling or less.

**TABLE 1:** Overview of study population characteristics (population size = 19,441) (Bern, Switzerland. 2025).

Variable	*n*	%	% Of current smokers
Total	19,441	100.0	​
Current smoking			
Non-smoker	14,985	74.9	​
Current-smoker	4,456	25.1	​
Sex			
Male	8,999	49.7	28.3
Female	10,442	50.3	21.9
Age			
15–24	2,023	11.9	26.1
25–34	2,105	16.7	29.0
35–44	2,969	17.9	28.4
45–54	3,686	18.0	25.9
55–64	4,027	17.7	24.9
65–74	3,270	12.6	18.7
75+	1,361	5.1	11.0
Migration background			
2nd or higher generation	1,443	8.4	34.4
1st generation	5,101	29.2	26.5
No migration background	12,897	62.4	23.1
Marital status			
Married^a^	10,854	48.8	20.5
Unmarried	8,587	51.2	29.4
Education			
Compulsory school or less	2,561	13.2	27.5
Secondary	8865	44.9	28.7
Tertiary	8,015	41.9	20.4
Employment status			
Employed	13,215	72.0	26.4
Not working^b^	5,915	25.9	20.1
Unemployed	311	2.0	39.1
Residence			
Urban	11,776	61.7	25.3
Peri-urban	4,121	21.8	24.3
Rural	3,544	16.6	25.1
Language region			
German	13,474	71.3	24.8
French	4,587	24.3	25.4
Italian	1,380	4.4	26.8
Alcohol use			
Abstinent	2'845	15.4	21.1
Occasional	12,044	62.9	23.9
Frequent	4,552	21.7	31.4
Drug use			
Never	14,323	70.2	18.0
More than 12 months ago	4,022	22.6	35.3
In the past 12 months	571	3.7	50.9
In the past 30 days	525	3.5	73.8

^a^ Including registered partnerships.

^b^ Outside of labour force, referring to retirees.

Most people were employed (68.0%), with 30.4% outside the labour force, and 1.6% unemployed. Most lived in urban areas (60%), and the German language region constituted nearly 70% of the sample, with 23.6% in the French-speaking region, and 7.1% in the Italian-speaking region.

Regarding modifiable health risk behaviours, 62.0% reported occasional alcohol use, 23.4% frequent use, and 14.6% abstinence, while 73.7% had never used drugs, and 26.3% reported past use of drugs.

### Association Between Current Smoking and Migration Background

In the multiple logistic regression model (see Supplementary Material 3, Supplementary Table S1), individuals with a 1st generation migration background (OR = 1.29, 95% CI: 1.18–1.40), and those with a 2nd generation or higher background (OR = 1.56, 95% CI: 1.37–1.78) had higher odds of current smoking compared to individuals with no migration background (reference group). In survey-weighted adjusted analyses restricted to migration background, 1st generation migration background had lower odds of current smoking than 2nd or higher generation migration backgrounds (OR 0.87, 95% CI 0.73–1.04); although this difference was not statistically significant (p = 0.120) (see Supplementary Material 3, Supplementary Table S2).

Design-based Wald tests indicated effect modification of the association between migration background and current smoking by age group (p < 0.001) and education (p = 0.043), but not by sex (p = 0.440) (Supplementary Material 2, Supplementary Table S2).

### Current Smoking and Migration Background Stratified by Sex

**FIGURE 1:**
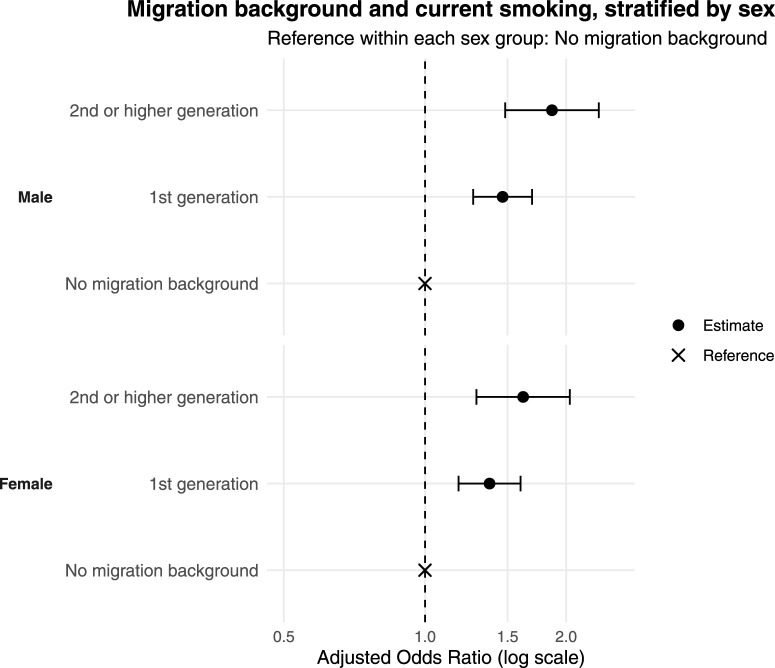
Migration background and current smoking, stratified by sex (Bern, Switzerland. 2025).

Sex-stratified estimates are presented to describe differences in current smoking by migration background, although formal interaction testing did not support effect modification by sex. Compared to those with no migrant background (reference group) (see Figure 1 and Table 2) both 1st generation (OR = 1.46, 95% CI: 1.27–1.69), and 2nd or higher-generation males showed higher odds of current smoking (OR = 1.87, 95% CI: 1.48–2.35). Among females, associations between migration background and current smoking were slightly weaker but remained significant (1st generation: OR = 1.37, 95% CI: 1.18–1.60; 2nd or higher-generation: OR = 1.62, 95% CI: 1.29–2.04) (see Figure 1).

**TABLE 2:** Stratified analysis: Sex, age, and education stratified adjusted odds ratios and 95% confidence interval for the association between migration background and smoking (population size = 19,441) (Bern, Switzerland. 2025).

Categories	Odds ratio	95% CI	p-value
**Sex**
**Male**			
2nd or higher generation	1.87	[1.48, 2.35]	<0.001
1st generation	1.46	[1.27, 1.69]	<0.001
No migration background	1.00	​	​
**Female**			
2nd or higher generation	1.62	[1.29, 2.04]	<0.001
1st generation	1.37	[1.18, 1.60]	<0.001
No migration background	1.00	​	​
**Age**			
**15–24**			
2nd or higher generation	3.04	[1.98, 4.68]	<0.001
1st generation	1.67	[1.09, 2.55]	0.018
No migration background	1.00	​	​
**25–34**			
2nd or higher generation	2.88	[1.91, 4.32]	<0.001
1st generation	1.78	[1.30, 2.43]	<0.001
No migration background	1.00	​	​
**35–44**			
2nd or higher generation	1.75	[1.16, 2.63]	0.008
1st generation	1.44	[1.14, 1.81]	0.002
No migration background	1.00	​	​
**45–54**			
2nd or higher generation	1.49	[1.05, 2.11]	0.027
1st generation	1.67	[1.34, 2.09]	<0.001
No migration background	1.00	​	​
**55–64**			
2nd or higher generation	0.95	[0.66, 1.35]	0.766
1st generation	0.94	[0.75, 1.17]	0.560
No migration background	1.00	​	​
**65–74**			
2nd or higher generation	2.06	[1.00, 4.26]	0.051
1st generation	1.00	[0.74, 1.33]	0.982
No migration background	1.00	​	​
**75+**			
2nd or higher generation	1.13	[0.26, 4.86]	0.868
1st generation	1.26	[0.75, 2.10]	0.381
No migration background	1.00	​	​
**Education**			
**Tertiary**			
2nd or higher generation	1.52	[1.16, 1.99]	0.003
1st generation	1.20	[1.02, 1.41]	0.029
No migration background	1.00	​	​
**Secondary**			
2nd or higher generation	1.90	[1.51, 2.39]	<0.001
1st generation	1.62	[1.39, 1.89]	<0.001
No migration background	1.00	​	​
**Compulsory school or less**			
2nd or higher generation	1.75	[1.05, 2.90]	0.031
1st generation	1.21	[0.87, 1.69]	0.252
No migration background	1.00	​	​

### Current Smoking and Migration Background Stratified by Age

**FIGURE 2:**
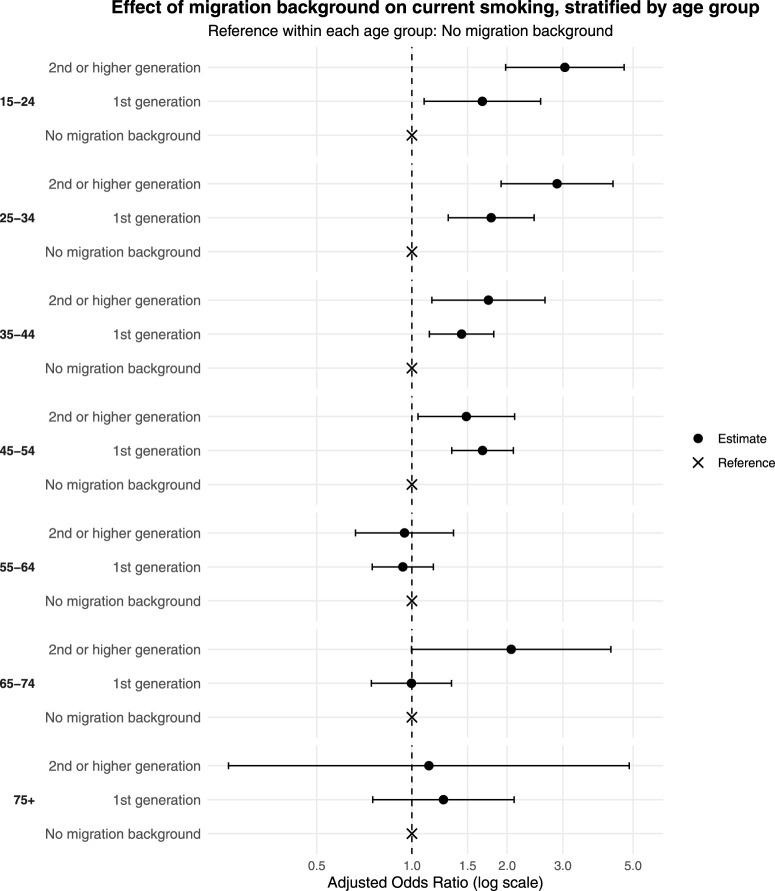
Effect of migration background on current smoking, stratified by age group (Bern, Switzerland. 2025).

Age-stratified analyses showed that the association between migration background and current smoking varied across age groups, with the most pronounced differences observed among younger adults. For people aged 15–24, those with a 2nd or higher-generation migration background had elevated odds of current smoking (OR = 3.04, 95% CI: 1.98–4.68), while 1st generation migration background also showed increased odds (OR = 1.67, 95% CI: 1.09–2.55) compared to people aged 15–24 with no migration background (see Figure 2 and Table 2). Nearly threefold higher odds of current smoking were observed in ages 25–34 with a 2nd or higher-generation migration background (OR = 2.88, 95% CI: 1.91–4.32) compared to those with no migration background in the same age group. Increased odds also were observed in people with 1st generation migration background aged 25–34 (OR = 1.78, 95% Cl: 1.30–2.43), as well as in age groups 35–44 (1st generation: OR = 1.44, 95% CI: 1.14–1.81; 2nd or higher-generation: OR = 1.75, 95% CI: 1.16–2.63). Elevated odds persisted for 1st generation migration background aged 45–54 (OR = 1.67, 95% CI: 1.34–2.09), and for 2nd or higher-generation in this group (OR = 1.49, 95% Cl: 1.05–2.11). No significant associations were observed among people aged 55 years or older.

### Current Smoking and Migration Background Stratified by Education

**FIGURE 3:**
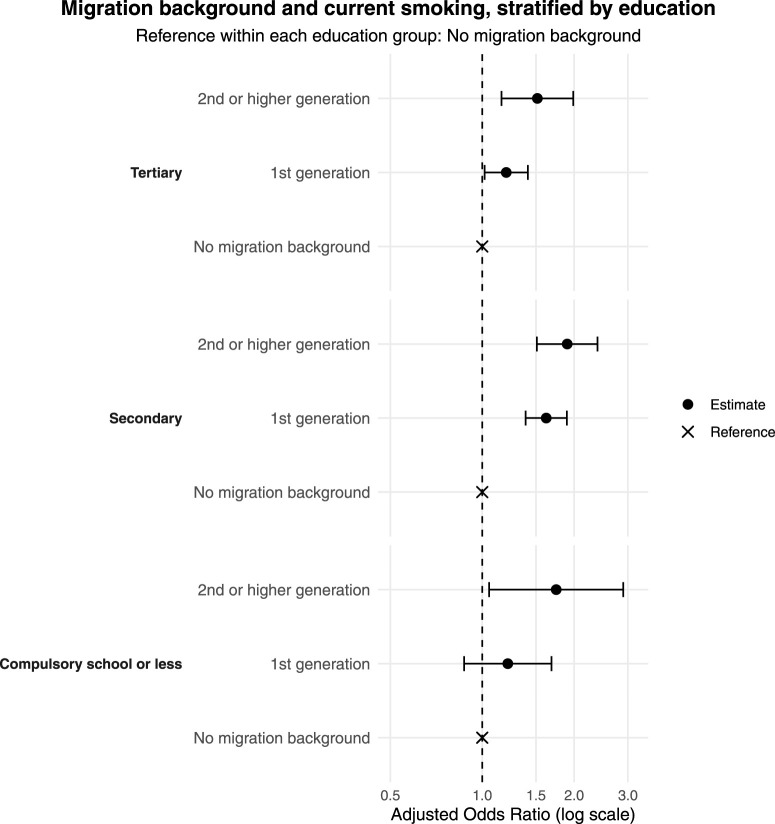
Migration background and current smoking, stratified by education (Bern, Switzerland. 2025).

Education-stratified analyses indicated that differences in smoking by migration background were more pronounced at lower levels of educational attainment. Compared to tertiary educated people with no migration background, tertiary-educatedwith a 2^nd^ or higher-generation migration background showed elevated odds (OR = 1.52, 95% CI: 1.16–1.99) (see Figure 3 and Table 2). In secondary education, both groups had significantly higher odds (1st generation: OR = 1.62, 95% CI: 1.39–1.89; 2nd or higher-generation: OR = 1.90, 95% CI: 1.51–2.39). High risk was also observed among 2nd or higher-generation migration background and compulsory education or less, who had 75% higher odds of current smoking (OR = 1.75, 95% CI: 1.05–2.90).

## Discussion

This study, using the most recent, official national data from the 2022 SHS, shows that people with migration backgrounds in Switzerland have consistently higher odds of current smoking than those without. This relationship persists even after accounting for a wide range of sociodemographic factors, as well as modifiable health risk behaviours alcohol and other drug use. In comparison to individuals with no migration background, elevated odds of current smoking were observed among both 1st and 2nd or higher-generation migration backgrounds. The highest odds ratios for the association between migration background and smoking were observed among younger adults (15–24 and 25–34 years), and among people with lower education attainment. These association were particularly pronounced odds for those with 2nd or higher-generation migration backgrounds within these age groups and among people with secondary education or less. Importantly, among individuals with a migration background, elevated odds also persisted among subgroups typically at lower risk for current smoking, such as tertiary-educated people, when compared to people without a migration background. This finding indicates that migration background is significantly associated with current smoking behaviour and intersects with sociodemographic indicators, such as age and education.

Our findings build on previous work by Wehrli et al. [7], which showed socially patterned smoking prevalence by Swiss citizenship status and education, but did not explore how migration background interacts with other social determinants [7]. While the overall regression models confirm higher odds of smoking among individuals with migration backgrounds, the stratified analyses help contextualise these associations in Switzerland. Specifically, they show that migration-related inequalities in current smoking are not confined to a single demographic group but are observable across multiple age and educational strata, with particularly pronounced differences among the 2^nd^ or higher generation migration backgrounds. Among 1st generation migration backgrounds, this may reflect continued exposure to smoking norms from countries of origin, stress related to migration and resettlement, or barriers to accessing prevention and cessation services in the host country [28]. At the same time, heterogeneity within this group, by country of origin, migration motive, and sociodemographic category, may contribute to more variable risk patterns [40, 41].

Our findings regarding people with 2nd generation migration backgrounds suggesting increased susceptibility to current smoking as a coping behaviour aligns with other research, as they experience distinct social stressors, including ethnic identity conflict, experiences of childhood disadvantage, while having less financial restrictions compared to people with 1st generation backgrounds [42–44]. Taken together, these results show that current smoking risk among people with migration backgrounds differs across generations.

At the same time, acculturation into the Swiss context where tobacco products are weakly regulated, remain widely available and relatively affordable could normalise smoking uptake through the sustained exposure to a permissive smoking environment [5, 45, 46]. These dynamics suggest that interventions should not only focus on those with a 1st generation migration background during resettlement, but must also be designed to address the unique challenges of 2nd generation migration background populations, who may be at heightened risk precisely because they straddle cultural expectations and social pressures from both the migrant and host communities [47, 48].

Age-stratified findings further enrich this picture. Elevated odds of current smoking were concentrated among younger adults with a migration backgrounds, with the highest odd ratios observed in those aged 15–24, and 25–34 with a 2nd or higher-generation migration background. This pattern reflects global evidence that greater experimentation and substance use initiation occurs in adolescence and early adulthood [49–51]. In contrast, differences in odds of being a current smoker were not significant among older adults (55+), which may largely be due to the smaller sample sizes manifesting in wider confidence intervals, and thus warranting cautious interpretation of effect sizes. The absence of marked differences among older adults may also reflect Switzerland’s overall decline in smoking prevalence in the recent decades (albeit more slowly than in neighbouring countries) [9, 52]. At the same time, selective survival bias may play a role: individuals who smoked heavily may be underrepresented in older age groups due to premature smoking-related mortality, which may reduce observed differences between those with migration backgrounds and those with no migration background. Together, these findings imply that inequalities emerge early and persist through critical life stages.

Similar associations between migration background and current smoking were observed in both men and women. Although smoking prevalence differed by sex in the overall model, formal interaction testing did not support effect modification by sex. These findings suggest that migration background is associated with smoking behaviour in a comparable way across sexes. Across many settings, smoking is more socially accepted among males, while female smoking has historically been constrained by cultural norms and family roles, contributing to lower reported prevalence but potentially greater barriers to cessation [53, 54]. Nonetheless, the persistence of elevated odds for current smoking among females with migration backgrounds in Switzerland suggests that traditional sex norms may be shifting, which may reflect international research suggesting that acculturation can lead to convergence towards host-country smoking norms, including shifts in sex-specific smoking patterns among migrant populations [55], emphasising the need for tobacco control interventions that explicitly integrate a sex- and gender-sensitive perspective across prevention, cessation, and communication approaches [56].

### Public Health and Policy Implications

Our results demonstrate that current smoking inequalities in Switzerland cannot be solely explained by traditional sociodemographic indicators, such as education and employment. Instead, migration background appears to shape how these inequalities manifest across population groups, with particularly elevated current smoking rates observed among people with migration backgrounds (1st and 2nd or higher generation) in younger age groups, and with lower education. The fact that tertiary education did not fully protect those with a migration background suggests that migration-related, structural and cultural barriers may outweigh the advantages usually conferred by higher education. This finding emphasises the need for outreach and community-based approaches in multiple languages that take into account the specific challenges faced by people with migration backgrounds. At the structural level, Switzerland’s relatively weak tobacco control environment must be strengthened through comprehensive advertising bans, higher taxation, and expanded cessation support. Without such measures, disparities are likely to persist or widen, particularly with the emergence of recent nicotine products (e.g., electronic cigarettes/vapes, nicotine pouches) [57].

### Strengths and Limitations

The major strength of this study is the use of the 2022 SHS, a large, nationally representative dataset, with robust weighting procedures, ensuring broad generalisability across Switzerland. The large sample size and even distribution across sexes and language regions provides sufficient statistical power to examine current smoking across key population subgroups [58].

The analytic sample population was large and broadly representative of the Swiss Health Survey population as well. Nevertheless, as with most survey-based studies, selection bias cannot be fully excluded, and excluded respondents differed from included respondents on some characteristics, particularly age and smoking status. These exclusions accounted for a small proportion of the sample.

Several limitations should be noted when interpreting these findings. First, although the overall sample size was large and nationally representative, and stratified analyses allowed us to identify current smoking patterns that would not be apparent in pooled analyses, some stratified analyses, particularly those involving 2nd or higher-generation migration backgrounds within specific age or education categories, were based on relatively small subgroup sizes. As a result, several subgroup-specific estimates may reflect reduced statistical precision. These findings should therefore be interpreted with caution, as they indicate greater uncertainty around the exact magnitude of the associations rather than the absence of an effect. We also suggest caution in regards to the educational attainment variable, which may be a less precise indicator among people with migration background (e.g., due to status incongruence following migration), and findings stratified by education should therefore be interpreted accordingly. Moreover, other subgroup sizes for certain variables (e.g., unemployed, and income categories) were even smaller, limiting the feasibility of further detailed stratification. Similarly small subgroups were observed for country of birth, which we excluded due to the high granularity and large number of country codes. A form of regional aggregation would have been necessary [59, 60] and was beyond the scope of this study. Furthermore, existing literature suggests that the country of origin *per se* may be less relevant than the contextual characteristics of the country, such as tobacco control policies (e.g., advertising bans, pricing, or taxation regulations), socio-cultural factors (e.g., social norms around shisha use), as well as the stage of the tobacco epidemic in the country people find themselves in [55, 61]. Notable differences can exist even between neighbouring countries, raising additional concerns about the validity of regional generalisations [62].

Second, current smoking status was measured by self-report and primarily captured cigarette use; although data on other nicotine products were available, their prevalence was very low (≤4%), precluding robust statistical analyses. As a result, findings would have likely underestimated differences in more recent products, such as heated tobacco products or e-cigarettes.

Third, migration background in this study was operationalised using generation status only (no migration background, 1st generation, 2nd or higher generation), based on the Swiss Federal Statistical Office typology. The SHS also lacks data on reasons for migration, or mobility (e.g., conflict vs. employment/education), acculturation level, language proficiency, the knowledge and attitudes towards tobacco smoking, or broader contextual factors, such as tobacco control environment in the country of origin, all of which may influence smoking behaviours [55, 63–66]. The role of cultural and social dynamics, such as tobacco smoking by immediate family and friends was also not in the SHS 2022 dataset [67]. As a result, simple aggregation by country or region of origin may obscure meaningful heterogeneity and introduce additional interpretive challenges. Future research would benefit from harmonised approaches that combine migration generation with contextual factors or country-origin tobacco policy and social norms. Other modifiable health-risk-related variables available in the SHS, such as body mass index, diet, and physical activity, were subject to reporting bias and missingness, or were not collected in a comprehensive or standardised manner suitable for multivariable adjustment. Thus, ensuring that these aspects were adequately included would have had significant methodological consequences, and was beyond the scope of the current study. Fourth, the survey was conducted only in German, French, and Italian, potentially underrepresenting individuals with limited language proficiency in these languages. Finally, the cross-sectional design prevents causal inference. Longitudinal studies with multiple time-points and further behavioural clustering (e.g., physical activity) are needed to better capture how tobacco and nicotine consumption evolves across generations in Switzerland.

### Conclusion

Migration background is a significant determinant of current smoking behaviour in Switzerland. Elevated odds were observed among people of both 1st and 2nd or higher-generation migration background, with particularly pronounced odds among younger adults, and especially amongst those with 2nd or higher generation migration backgrounds. Higher odds persisted even among groups typically at lower risk for smoking, such as 2nd or higher-generation with tertiary education, highlighting the need for tobacco control strategies that go beyond socioeconomic status to address migration background specific factors. The observed patterns have important implications for public health practice and policy, suggesting a need for culturally adapted and equity-focused interventions, combined with national tobacco control policies that may help reduce smoking prevalence.
