# Supplementary material for: Sociodemographic Differences in Smoking Behaviours by Migration Background: Insights From the National Swiss Health Survey
Source: Int J Public Health. 2026 Apr 20;71:1609268. doi: 10.3389/ijph.2026.1609268 (PMC13136040; doi:10.3389/ijph.2026.1609268)
Supplement: Supplementary file 1 [file Supplementaryfile1.docx]

International Journal of Public Health

Sociodemographic differences in smoking behaviours among people with a migration background: Insights from the national Swiss Health Survey

Supplementary Material 1

Description of coded variables

Table S1: Variables used for the definition of first- and second-generation migrants, schema by the Federal Statistical Office

| Place of birth | Nationality | Place of birth of the parents | | |
| --- | --- | --- | --- | --- |
|  |  | 2 in Switzerland | 1 in Switzerland and 1 abroad | 2 abroad |
| Switzerland | Swiss at birth | 0 | 0 | II |
|  | Swiss by naturalisation | 0 | II | II |
|  | Foreigner | 0 | II | II |
| Abroad | Swiss at birth | 0 | 0 | I |
|  | Swiss by naturalisation | I | I | I |
|  | Foreigner | I | I | I |

I am a Population with a migration background, 1st generation
II Population with a migration background, 2nd generation
0 Population without a migration background

Table S2: Overview and description of variables included in analyses

| **Outcome** | **Final variable name** | **Final coding** | **Original question (translated from German (Switzerland) / Comments** |
| --- | --- | --- | --- |
| Current tobacco consumption | TABAC3 | 1, 2 = never smoker. former smoker  3 = current smoker | Do you smoke, even if only occasionally?  (Including heated tobacco products such as IQOS, but excluding e-cigarettes.)   - Yes - No   +  If non-smoker: Have you ever smoked for more than 6 months?   - Yes - No |
| **Explanatory/**  **Exposure variables** | **Variable name** | **Final coding** | **Original question (translated from German (Switzerland) / Comments** |
| Age | alter7 | 1= 15- 24 years  2= 25-34 years  3= 35-44 years  4= 45-54 years  5= 55-64 years  6= 65-74 years  7= 75+ years | Based on registered birthdate |
| Sex | sex | 1= Male  2= Female | Who are you?  Male / Female |
| Civil Status | maritalstatus | 1= Married*  2= Unmarried  *including registered partnerships | Marital status according to register  Married included registered partnerships  Recoding:  1= Married  2= Unmarried  3= Widowed  4= Divorced  5= Unmarried  6= In registered partnership  7= Dissolved partnership¨  Married, including registered partnerships:  1 = 2, 6  Unmarried:  2= 1, 3, 4, 5, 7 |
| Migration Background | statmigr | 1 = Population without migrationbackground  2 = Population with migrationbackground 1. generation  3 = Population with migration background 2. generation or higher generations | Utilised variables:   - TSODE97 – Nationality at birth - NATION3 – Current nationality and naturalisation status - COUNTRYIDOFBIRTH – Country of birth (according to register) - TSODE103 – Father’s country of birth - TSODE104 – Mother’s country of birth   See Table 2 below for typology used by the Federal Statistics Office (FSO). |
| Residence location | stala | 1 = Urban  2 =Intermediate (dense peri-urban areas and rural centres)  3 = Rural | Recoding based on ‘[The Spatial Divisions of Switzerland](https://www.bfs.admin.ch/bfs/de/home/grundlagen/raumgliederungen.html)’ by the FSO, as of 1 January 2014 |
| Language region | sprache | 1 = German  2 = French  3 = Italian | The classification of municipalities into a language region is based on the document ‘[The Spatial Divisions of Switzerland](https://www.bfs.admin.ch/bfs/de/home/grundlagen/raumgliederungen.html)’, which in turn relies on the census and the structural survey.  This index takes into account the municipal language of the place where the respondent lives. Accordingly, the ‘French-speaking Switzerland’ region includes not only the cantons of Vaud and Geneva but also various municipalities in the cantons of Fribourg, Valais, Jura and Bern (Bernese Jura). The language indicated in this index does not always correspond to the language in which the interview was conducted (INTSPRACH). |
| Education | AUSBILD3 | 1 = Compulsory school or less  2 = Secondary  3 = Tertiary | Highest level of education completed  Categorised according to milestones in the Swiss education system:   - Compulsory school or less – primary and lower secondary education (mandatory 11 years). - Secondary II – upper secondary level, including vocational training, apprenticeships, or general education (e.g. baccalaureate). - Tertiary – education beyond upper secondary, such as universities or universities of applied sciences. |
| Employment status | erwerb | 1= not working/not in labour force  2= Unemployed  3= Employed person | Utilised varibales:   - TARSI49 - Paid employment during the last week - TARSI50 – Unpaid work in a family business during the last week - TARSI51 – Employed, although did not work during the last week - TARSI70 – Non-employed: availability in the event of a job offer   Category label:  1 = predominantly retired persons |
| Alcohol Consumption | TALKO15 | 1 = never, abstinent  2 = occasional use. (weekly to less than once per month)  3 = frequent use, (daily or several times per week) | How often do you usually drink alcoholic beverages (e.g. beer, wine, liqueur, aperitif, spirits, schnapps)?    --------------------------------------------- Original categories:  1 – 3 times or more per day → 03820  2 – Twice per day (with meals) → 03820  3 – Once per day → 03820  4 – Several times per week → 03820  5 – 1–2 times per week → 03820  6 – 1–3 times per month → 03820  7 – Less than once per month → 03861  8 – Never, abstinent  We wanted a parsimonious 3-level variable that still distinguishes heavy/frequent drinking from more occasional/social drinking, while also keeping a clear abstinent reference group.  It reduced sparse categories (like “3 times per day” which are rare) and made regression results more interpretable.  Recoded:  1 = 8  2 = 5, 6, 7  3 = 1, 2, 3, 4 |
| Drug consumption | DROGCONS | 1 = never tried drugs  3 = more than 12 months ago  4 = in the past 12 months  5 = in the past 30 days | TDROG01:  Have you ever in your life taken drugs, e.g. hash, cocaine or other drugs?   - Yes - No (1)   Follow-up utilised variables:  HACHCONS – Cannabis use  Have you ever used/taken cannabis (hash/marijuana)?   - Yes - No (-> next drug)   +  If yes, Have you used/taken cannabis (hash/marijuana) in the past:   - 12 months   - Yes (-> 30 days)   - No (3) - 30 days   - Yes (5) (-> in the past 30 days…)   - No (4)   If yes 30 days, in the past 30 days, how often have used/taken cannabis (hash/marijuana)?   - Daily, or almost daily (5) - Several time per week (5) - Once per week (5) - Even less (5)   +  DURECONS – Use of hard drugs  Repeated question structure for each cocaine, heroin, ecstasy and “other drugs” seperately.  For instance cocaine:  Have you ever used/taken cocaine?   - Yes - No   +  If yes, Have you used/taken cocaine in the past:   - 12 months   - Yes (-> 30 days)   - No (3) - 30 days   - Yes (5) (-> in the past 30 days…)   - No (4)   If yes 30 days, in the past 30 days, how often have used/taken cocaine?   - Daily, or almost daily (5) - Several time per week (5) - Once per week (5) - Even less (5)   For some reason coded without “2”.  Drug consumption variable (DROGCONS) was derived using above questions on cannabis (HACHCONS) and other hard drugs (DURECONS).  DROGCONS:  1 = if No TDROG01  3 = HACHCONS Yes, then No (3), DURECONS Yes, then No (3)  4 = HACHCONS Yes, Yes, then No (4), DURECONS Yes, Yes, then No (4)  5 = HACHCONS Yes, Yes, Yes (5), DURECONS Yes, Yes, Yes (5) |
