# Supplementary material for: Sociodemographic Differences in Smoking Behaviours by Migration Background: Insights From the National Swiss Health Survey
Source: Int J Public Health. 2026 Apr 20;71:1609268. doi: 10.3389/ijph.2026.1609268 (PMC13136040; doi:10.3389/ijph.2026.1609268)
Supplement: Supplementary file 2 [file Supplementaryfile3.docx]

International Journal of Public Health

Sociodemographic differences in smoking behaviours among people with a migration background: Insights from the national Swiss Health Survey

Supplementary Material 3

Table S1. Associations between smoking and sociodemographic factors among SHS participants using survey-weighted multivariable logistic regression (n=19,441)

| Category | Odds Ratio | 95% Confidence Interval | P-value* |
| --- | --- | --- | --- |
| **Sex** | | | |
| Male | 1 (ref) |  |  |
| Female | 0.85 | [0.77, 0.93] | <0.001 |
| **Age** | | | |
| 15-24 | 1 (ref) |  |  |
| 25-34 | 1.68 | [1.38, 2.06] | <0.001 |
| 35-44 | 2.06 | [1.69, 2.51] | <0.001 |
| 45-54 | 1.92 | [1.58, 2.34] | <0.001 |
| 55-64 | 1.92 | [1.59, 2.32] | <0.001 |
| 65-74 | 1.58 | [1.28, 1.95] | <0.001 |
| 75+ | 0.91 | [0.69, 1.20] | 0.513 |
| **Marital status** | | | |
| Married/Registered Partnership | 1 (ref) |  |  |
| Unmarried | 1.45 | [1.32, 1.60] | <0.001 |
| **Migration background** | | | |
| No migration background | 1 (ref) |  |  |
| 1st generation | 1.42 | [1.28, 1.58] | <0.001 |
| 2nd or higher generation | 1.75 | [1.49, 2.06] | <0.001 |
| **Education** | | | |
| Compulsory school or less | 1 (ref) |  |  |
| Secondary | 0.89 | [0.77, 1.03] | 0.118 |
| Tertiary | 0.45 | [0.39, 0.53] | <0.001 |
| **Employment status** | | | |
| Employed | 1 (ref) |  |  |
| Not working | 0.91 | [0.80, 1.04] | 0.170 |
| Unemployed | 1.51 | [1.10, 2.07] | 0.011 |
| **Residence** | | | |
| Urban | 1 (ref) |  |  |
| Peri-urban | 1.07 | [0.96, 1.20] | 0.234 |
| Rural | 1.13 | [1.00, 1.27] | 0.053 |
| **Language region** | | | |
| German | 1 (ref) |  |  |
| French | 0.92 | [0.83, 1.01] | 0.095 |
| Italian | 1.10 | [0.95, 1.27] | 0.216 |
| **Alcohol use** | | | |
| Abstinent | 1 (ref) |  |  |
| Occasional | 1.10 | [0.96, 1.26] | 0.156 |
| Frequent | 1.62 | [1.38, 1.90] | <0.001 |
| **Drug use** | | | |
| Never | 1 (ref) |  |  |
| more than 12 months ago | 2.45 | [2.20, 2.72] | <0.001 |
| in the past 12 months | 4.69 | [3.76, 5.85] | <0.001 |
| in the past 30 days | 12.33 | [9.54, 15.95] | <0.001 |

*Note:* * *P-values are based on design-based Wald tests from survey-weighted multivariable logistic regression models.*

Figure S1: Adjusted odds ratios and 95% confidence interval for current smoking, based on a survey-weighted multivariable logistic regression model (n=19,441),

Lower risk of current smoking

Higher risk of current smoking


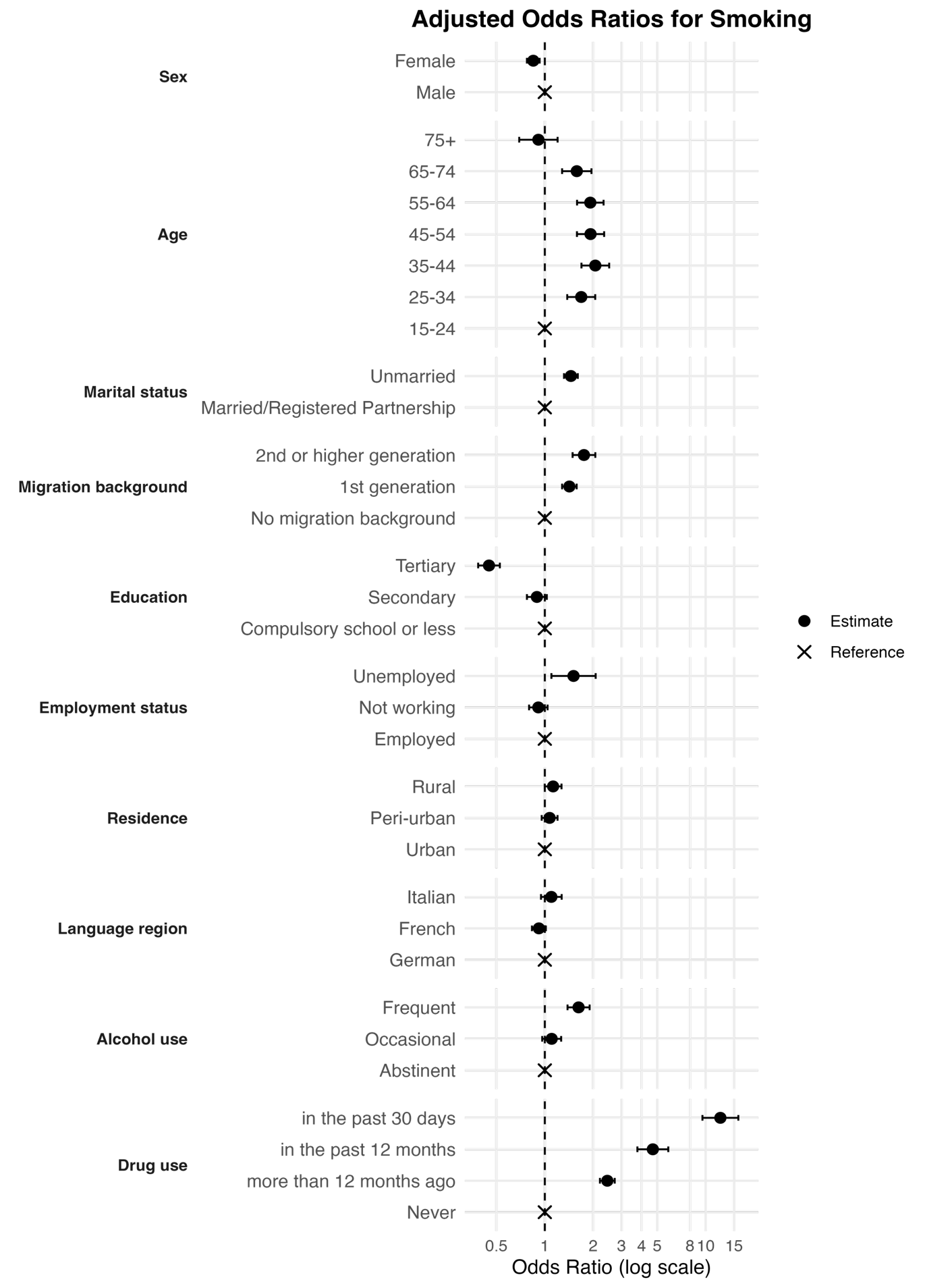


Associations between current smoking status and sociodemographic factors, alcohol and drug use

In the multivariable logistic regression model, migration background was significantly associated with smoking status: individuals with a 1^st^ generation migration background had higher odds of current smoking (OR = 1.42, 95% CI: 1.28–1.49), while those with a 2^nd^ generation or higher background exhibited even greater odds (OR = 1.75, 95% CI: 1.48–2.06) compared to individuals with no migration background (reference group). Age was also strongly associated with current smoking. Compared to individuals aged 15–24 years (reference group), those aged 25–64 had significantly higher odds of current smoking, with the highest association observed among those aged 35–44 (OR = 2.06, 95% CI: 1.69–2.51). No significant association between age and current smoking status was observed among participants aged 75 and older (OR = 0.91, 95% CI: 0.69–1.20). In line with our hypotheses, females had significantly lower odds of smoking compared to males (OR = 0.85, 95% CI: 0.77–0.93) (see Table S1 & Figure S1).

Unmarried individuals had greater odds of current smoking relative to those who were married or in a registered partnership (reference group) (OR = 1.45, 95% CI: 1.32–1.60).

Higher educational attainment showed an inverse association with current smoking. Compared to those with compulsory schooling or less (reference group), individuals with a tertiary education were substantially less likely to currently smoke (OR = 0.45, 95% CI: 0.39–0.53). Employment status was also a significant factor: unemployed individuals had higher odds of current smoking compared to those who were employed (OR = 1.51, 95% CI: 1.10–2.07), while those not in the labour force had slightly lower odds (OR = 0.91, 95% CI: 0.80–1.04) compared to employed individuals.

No significant differences in current smoking status were observed by residential setting (peri-urban: OR = 1.07, 95% CI: 0.96–1.20; rural: OR = 1.13, 95% CI: 1.00–1.27; reference: urban). No significant differences were similarly observed by language region when compared to residents in the German-speaking region (reference group) (Italian-speaking OR = 1.10, 95% CI: 0.95.–1.27; French-speaking region OR = 0.92, 95% CI: 0.82–1.01).

Compared to abstainers (reference group), occasional drinkers had slightly higher odds of current smoking though not significant (OR = 1.10, 95% CI: 0.96–1.26). Frequent drinkers had markedly elevated odds (OR = 1.62, 95% CI: 1.38–1.90). Lastly, drug use showed a strong association with current smoking: individuals who reported drug use more than 12 months ago (OR = 2.45, 95% CI: 2.20–2.72), in the past 12 months (OR = 4.69, 95% CI: 3.76–5.85), and in the past 30 days (OR = 12.33, 95% CI: 9.54–15.95) had progressively higher odds of current smoking compared to those who had never used drugs (reference group).

Table S2: Adjusted comparison between first- and second-generation migrants

| Migration background comparison | Adjusted odds ratio (OR) | 95% confidence interval | p-value |
| --- | --- | --- | --- |
| First-generation vs. second-generation migrants | 0.87 | 0.73–1.04 | 0.120 |

Note: Odds ratios were estimated using survey-weighted multivariable logistic regression adjusted for age group, sex, education, employment status, marital status, residence type, language region, alcohol consumption, and drug use. Second-generation migrants were specified as the reference category.
