# Supplementary material for: Sociodemographic Differences in Smoking Behaviours by Migration Background: Insights From the National Swiss Health Survey
Source: Int J Public Health. 2026 Apr 20;71:1609268. doi: 10.3389/ijph.2026.1609268 (PMC13136040; doi:10.3389/ijph.2026.1609268)
Supplement: Supplementary file 3 [file Supplementaryfile2.docx]

International Journal of Public Health

Sociodemographic differences in smoking behaviours among people with a migration background: Insights from the national Swiss Health Survey

Supplementary Material 2:

Table S1: Included vs Excluded: Study Population Overview

Total N = 21'930, Included = 19'441, Excluded = 2'489

Distribution of key socio-demographic variables and modifiable chronic disease risk behaviours between participants included (n = 19,441) and excluded (n = 2,489) in the analytic sample. Statistical differences were tested using Mann-Whitney-U/Wilcoxon rank-sum tests (for ordinal/numeric variables) and Chi-square tests (for nominal variables) (see Note below for test per variable).

| Category | Included (n) | Included (%) of Total | Excluded (n) | Excluded (%) of Total | P-value | Effect |
| --- | --- | --- | --- | --- | --- | --- |
| Total 21,930 | | | | |  |  |
|  | 19,441 | 88.7 | 2,489 | 11.3 |  |  |
| **Smoking status**^1^ | | | | | <0.001 | Cramér's V = 0.070 [0.059, 1.000] |
| Non-smoker | 14,985 | 68.3 | 2,137 | 9.7 |  |  |
| Smoker | 4,456 | 20.3 | 340 | 1.6 |  |  |
| Missing values | 0 | 0.0 | 12 | 0.1 |  |  |
| **Sex**^1^ | | | | | 0.646 | Cramér's V = 0.000 [0.000, 1.000] |
| Male | 8,999 | 41.0 | 1,140 | 5.2 |  |  |
| Female | 10,442 | 47.6 | 1,349 | 6.2 |  |  |
| **Age**^2^ | | | | | <0.001 | r = 0.353 [0.340, 0.370] |
| 15–24 | 2,023 | 9.2 | 93 | 0.4 |  |  |
| 25–34 | 2,105 | 9.6 | 75 | 0.3 |  |  |
| 35–44 | 2,969 | 13.5 | 153 | 0.7 |  |  |
| 45–54 | 3,686 | 16.8 | 183 | 0.8 |  |  |
| 55–64 | 4,027 | 18.4 | 179 | 0.8 |  |  |
| 65–74 | 3,270 | 14.9 | 153 | 0.7 |  |  |
| 75+ | 1,361 | 6.2 | 1,653 | 7.5 |  |  |
| **Marital status**^1^ | | | | | 0.665 | Cramér's V = 0.000 [0.000, 1.000] |
| Married/Registered Partnership | 10,854 | 49.5 | 1,401 | 6.4 |  |  |
| Unmarried | 8,587 | 39.2 | 1'088 | 5.0 |  |  |
| **Migration background**^1^ | | | | | <0.001 | Cramér's V = 0.066 [0.055, 1.000] |
| No migration background | 12,897 | 58.8 | 1,246 | 5.7 |  |  |
| 1st generation | 5,101 | 23.3 | 391 | 1.8 |  |  |
| 2nd or higher generation | 1,443 | 6.6 | 27 | 0.1 |  |  |
| Missing values | 0 | 0.0 | 825 | 3.8 |  |  |
| **Education**^2^ | | | | | <0.001 | r = 0.146 [0.130, 0.160] |
| Compulsory school or less | 2,561 | 11.7 | 698 | 3.2 |  |  |
| Secondary | 8,865 | 40.4 | 1,092 | 5.0 |  |  |
| Tertiary | 8,015 | 36.5 | 555 | 2.5 |  |  |
| Missing values | 0 | 0.0 | 144 | 0.7 |  |  |
| **Employment status**^1^ | | | | | <0.001 | Cramér's V = 0.307 [0.295, 1.000] |
| Employed | 13,215 | 60.3 | 566 | 2.6 |  |  |
| Not working | 5,915 | 27.0 | 1,902 | 8.7 |  |  |
| Unemployed | 311 | 1.4 | 8 | 0.0 |  |  |
| Missing values | 0 | 0.0 | 13 | 0.1 |  |  |
| **Residence**^1^ | | | | | <0.001 | Cramér's V = 0.037 [0.024, 1.000] |
| Urban | 11,776 | 53.7 | 1,645 | 7.5 |  |  |
| Peri-urban | 4,121 | 18.8 | 484 | 2.2 |  |  |
| Rural | 3,544 | 16.2 | 360 | 1.6 |  |  |
| **Language region**^1^ | | | | | 0.076 | Cramér's V = 0.012 [0.000, 1.000] |
| German | 13,474 | 61.4 | 1,777 | 8.1 |  |  |
| French | 4,587 | 20.9 | 557 | 2.5 |  |  |
| Italian | 1,380 | 6.3 | 155 | 0.7 |  |  |
| **Alcohol use**^2^ | | | | | <0.001 | r = 0.031 [0.020, 0.050] |
| Abstinent | 2,845 | 13.0 | 686 | 3.1 |  |  |
| Occasional | 12,044 | 54.9 | 1,089 | 5.0 |  |  |
| Frequent | 4,552 | 20.8 | 691 | 3.2 |  |  |
| Missing values | 0 | 0.0 | 23 | 0.1 |  |  |
| **Drug use**^2^ | | | | | <0.001 | r = 0.067 [0.060, 0.080] |
| Never | 14,323 | 65.3 | 710 | 3.2 |  |  |
| more than 12 months ago | 4,022 | 18.3 | 65 | 0.3 |  |  |
| in the past 12 months | 571 | 2.6 | 13 | 0.1 |  |  |
| in the past 30 days | 525 | 2.4 | 10 | 0.0 |  |  |
| Missing values | 0 | 0.0 | 1,691 | 7.7 |  |  |

Note: ^1^ Pearson Chi-square test, ^2^ Mann-Whitney-U / Wilcoxon rank-sum

To evaluate potential selection bias arising from missing covariate data, we statistically compared included and excluded participants across all variables used in the main analyses. For continuous and ordinal variables (age, education, alcohol use, and drug use), group differences were assessed using the Mann–Whitney U / Wilcoxon rank-sum test with r as an effect size measure. For nominal variables (sex, marital status, migration background, employment status, residence, language region, and current smoking status), Pearson’s Chi-square tests were applied, and Cramér’s V was computed to quantify the strength of association.

Confidence intervals for Cramér’s V were not calculated, as standard analytical methods are limited for multi-category contingency tables; reported values therefore reflect point estimates derived directly from the Chi-square statistic. Effect sizes were interpreted according to conventional benchmarks: r values of 0.1, 0.3, and 0.5 indicate small, moderate, and large differences, respectively; Cramér’s V values of approximately 0.06, 0.17, and 0.29 indicate small, medium, and large associations.

Older participants (75+) constituted 66% (n = 1653) of the total excluded sample (n=2489), producing a large age difference between included and excluded participants (r = 0.35, p < 0.001). Excluded participants also tended to have lower educational attainment (compulsory schooling) (r = 0.15, p < 0.001). They reported less frequent alcohol consumption, and were less likely to report recent drug use, although these latter associations were small in magnitude (r = 0.03, p < 0.001, and r = 0.07, p < 0.001 respectively). Chi-square tests showed small to moderate differences. Excluded participants were more likely to have no migration background (V = 0.07, p < 0.001), more likely to be outside of the labour force (V = 0.31, p < 0.001), and slightly more likely to reside in urban areas (V = 0.04, p < 0.001). No meaningful differences were observed for sex (V ≈ 0.00, p = 0.646), marital status (V ≈ 0.00, p = 0.665), or language region (V = 0.01, p = 0.076). Current smoking status also differed slightly (V = 0.07, p < 0.001), with excluded participants being more likely to be non-smokers.

Table S2: Design-based Wald tests for effect modification

Survey-weighted models with interaction terms

| Interaction | Wald F | df | p-value | N |
| --- | --- | --- | --- | --- |
| Migration background × Sex | 0.82 | 2 | 0.440 | 19,441 |
| Migration background × Age group | 3.31 | 12 | <0.001 | 19,441 |
| Migration background × Education | 2.46 | 4 | 0.043 | 19,441 |
