# Supplementary material for: Sociodemographic Differences in Smoking Behaviours by Migration Background: Insights From the National Swiss Health Survey
Source: Int J Public Health. 2026 Apr 20;71:1609268. doi: 10.3389/ijph.2026.1609268 (PMC13136040; doi:10.3389/ijph.2026.1609268)
Supplement: Supplementary file 4 [file Supplementaryfile4.docx]

International Journal of Public Health

Sociodemographic differences in smoking behaviours by migration background: Insights from the national Swiss Health Survey

Supplementary Material 4

RStudio Code book:

Table of Contents

[Step 1: 2](#_Toc212736484)

[Clean data set and missings analysis 2](#_Toc212736485)

[Step 2: 11](#_Toc212736486)

[Multivariable logistic regression analysis: 11](#_Toc212736487)

[Step 3: 19](#_Toc212736488)

[Stratified Analyses 19](#_Toc212736489)

[Stratified analysis 1: sex 19](#_Toc212736490)

[Stratified analysis 2: age 24](#_Toc212736491)

[Stratified analysis 3: education 27](#_Toc212736492)

Step 1:

Clean data set and missings analysis

# Load packages

# -----------------------------

install.packages("tidyverse")

install.packages("gtsummary")

install.packages("survey")

library(tidyverse)

library(gtsummary)

library(broom)

library(stringr)

library(tibble)

library(purrr)

library(dplyr)

library(gt)

library(ggplot2)

library(scales)

library(forcats)

library(haven)

library(survey)

# -----------------------------

# Import and read raw dataset

# -----------------------------

tel22_ch <- read_dta("Documents/PhD/SGB analysis/Data SGB2022/tel22_ch.dta")

indic22_ch <- read_dta("Documents/PhD/SGB analysis/Data SGB2022/indic22_ch.dta")

# -----------------------------

# Merge data

# -----------------------------

merged_data <- merge(indic22_ch, tel22_ch, by = "idno", all.x = TRUE)

# Keep variables INCLUDING weight

merged_data <- merged_data[, c(

"idno", "wght", "alter", "TABAC3", "sex", "maritalstatus", "statmigr", "stala",

"sprache", "AUSBILD3", "erwerb", "TALKO15", "drogcons"

)]

# -----------------------------

# Step 1: Clean missing values

# -----------------------------

invalid_codes <- c(-1, -2, -3, -4, -5, -6, -8)

vars_to_clean <- c(

"alter", "TABAC3", "sex", "maritalstatus", "statmigr", "stala",

"sprache", "AUSBILD3", "erwerb", "TALKO15", "drogcons"

)

merged_data[vars_to_clean] <- lapply(merged_data[vars_to_clean], function(x) {

x <- as.numeric(as.character(x))

x[x %in% invalid_codes] <- NA

return(x)

})

# Clean weight variable

merged_data$wght <- as.numeric(as.character(merged_data$wght))

merged_data$wght[!is.finite(merged_data$wght) | merged_data$wght <= 0] <- NA

# -----------------------------

# Step 2: Create derived variables

# -----------------------------

merged_data <- merged_data %>%

mutate(

# Age groups

alter7 = case_when(

alter >= 15 & alter <= 24 ~ "15–24",

alter >= 25 & alter <= 34 ~ "25–34",

alter >= 35 & alter <= 44 ~ "35–44",

alter >= 45 & alter <= 54 ~ "45–54",

alter >= 55 & alter <= 64 ~ "55–64",

alter >= 65 & alter <= 74 ~ "65–74",

alter >= 75 ~ "75+",

TRUE ~ NA_character_

),

# Smoking outcome (numeric for weighted calculations)

smoker_binary_num = case_when(

TABAC3 == 3 ~ 1,

TABAC3 %in% c(1, 2) ~ 0,

TRUE ~ NA_real_

),

# Smoking outcome (factor for display)

smoker_binary = factor(

smoker_binary_num,

levels = c(0, 1),

labels = c("Non-smoker", "Current-smoker")

),

# Marital status

maritalstatus_recoded = case_when(

maritalstatus %in% c(2, 6) ~ 1,

maritalstatus %in% c(1, 3, 4, 5, 7) ~ 2,

TRUE ~ NA_real_

),

# Alcohol use

alcohol_use = case_when(

TALKO15 == 8 ~ 1,

TALKO15 %in% c(5, 6, 7) ~ 2,

TALKO15 %in% c(1, 2, 3, 4) ~ 3,

TRUE ~ NA_real_

),

# Drug use

drug_use = case_when(

drogcons == 5 ~ 4,

drogcons == 4 ~ 3,

drogcons == 3 ~ 2,

drogcons == 1 ~ 1,

TRUE ~ NA_real_

)

)

# -----------------------------

# Step 3: Convert variables to factors with readable labels

# -----------------------------

merged_data <- merged_data %>%

mutate(

alter7 = factor(alter7, levels = c("15–24", "25–34", "35–44", "45–54", "55–64", "65–74", "75+")),

sex = factor(sex, levels = c(1, 2), labels = c("Male", "Female")),

maritalstatus_recoded = factor(maritalstatus_recoded, levels = c(1, 2),

labels = c("Married*", "Unmarried")),

statmigr = factor(statmigr, levels = c(3, 2, 1),

labels = c("2nd or higher generation", "1st generation", "No migration background")),

stala = factor(stala, levels = c(1, 2, 3),

labels = c("Urban", "Peri-urban", "Rural")),

sprache = factor(sprache, levels = c(1, 2, 3),

labels = c("German", "French", "Italian")),

erwerb = factor(erwerb, levels = c(3, 1, 2),

labels = c("Employed", "Not working**", "Unemployed")),

AUSBILD3 = factor(AUSBILD3, levels = c(1, 2, 3),

labels = c("Compulsory school or less", "Secondary", "Tertiary")),

alcohol_use = factor(alcohol_use, levels = c(1, 2, 3),

labels = c("Abstinent", "Occasional", "Frequent")),

drug_use = factor(drug_use, levels = c(1, 2, 3, 4),

labels = c("Never", "more than 12 months ago", "in the past 12 months", "in the past 30 days"))

)

Screenshot merged and relabelled data set:


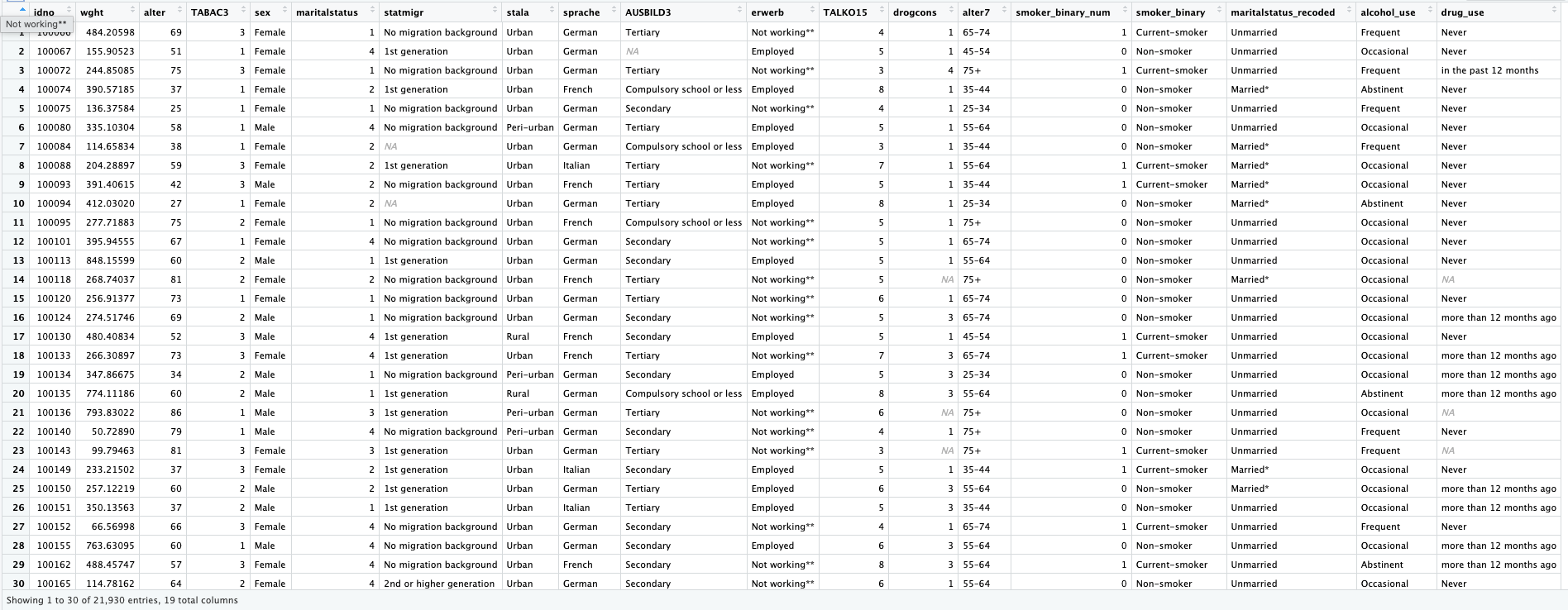


# --- Overview Table of Study Population ---

# Define the exact complete-case analytic sample used in model1

# ============================================================

model_vars <- c(

"smoker_binary_num", "sex", "alter7", "maritalstatus_recoded",

"statmigr", "AUSBILD3", "erwerb", "stala", "sprache",

"alcohol_use", "drug_use", "wght"

)

analysis_data <- merged_data %>%

filter(complete.cases(across(all_of(model_vars)))) %>%

droplevels()

cat("N in complete-case analytic sample =", nrow(analysis_data), "\n")

# Survey design object

design_main <- svydesign(

ids = ~1,

weights = ~wght,

data = analysis_data

)

# Helper function:

# Creates one Table 1 block with

# - unweighted n

# - weighted %

# - weighted % of current smokers

make_weighted_block <- function(data, design, var, var_label) {

# unweighted counts

counts <- data %>%

count(.data[[var]], name = "n") %>%

rename(Category = .data[[var]]) %>%

mutate(Category = as.character(Category))

# weighted %

wt_dist <- svytable(as.formula(paste0("~", var)), design)

wt_pct <- prop.table(wt_dist) * 100

wt_pct_df <- tibble(

Category = names(wt_pct),

Percent = as.numeric(wt_pct)

)

# weighted % of current smokers within each category

smoker_df <- split(data, data[[var]])

smoker_pct_df <- purrr::map_dfr(names(smoker_df), function(cat_name) {

ds_sub <- subset(design, get(var) == cat_name)

# weighted smoking prevalence within category

prev <- tryCatch(

coef(svymean(~smoker_binary_num, ds_sub, na.rm = TRUE))[1] * 100,

error = function(e) NA_real_

)

tibble(

Category = cat_name,

Smokers_Percent = prev

)

})

out <- counts %>%

left_join(wt_pct_df, by = "Category") %>%

left_join(smoker_pct_df, by = "Category") %>%

mutate(

Variable = var_label,

Percent = round(Percent, 1),

Smokers_Percent = round(Smokers_Percent, 1)

) %>%

select(Variable, Category, n, Percent, Smokers_Percent)

out

}

# Build Table 1 blocks

# Smoking block (special case)

smoking_counts <- analysis_data %>%

count(smoker_binary, name = "n") %>%

rename(Category = smoker_binary)

smoking_prev <- svymean(~smoker_binary_num, design_main, na.rm = TRUE)

smoker_prev <- coef(smoking_prev)[1] * 100

smoking_block <- tibble(

Variable = "Current smoking",

Category = c("Non-smoker", "Current-smoker"),

n = smoking_counts$n[match(c("Non-smoker", "Current-smoker"), smoking_counts$Category)],

Percent = round(c(100 - smoker_prev, smoker_prev), 1),

Smokers_Percent = c(NA, NA)

)

# Other variable blocks

sex_block <- make_weighted_block(analysis_data, design_main, "sex", "Sex")

age_block <- make_weighted_block(analysis_data, design_main, "alter7", "Age")

mig_block <- make_weighted_block(analysis_data, design_main, "statmigr", "Migration background")

marital_block <- make_weighted_block(analysis_data, design_main, "maritalstatus_recoded", "Marital status")

edu_block <- make_weighted_block(analysis_data, design_main, "AUSBILD3", "Education")

emp_block <- make_weighted_block(analysis_data, design_main, "erwerb", "Employment status")

res_block <- make_weighted_block(analysis_data, design_main, "stala", "Residence")

lang_block <- make_weighted_block(analysis_data, design_main, "sprache", "Language region")

alc_block <- make_weighted_block(analysis_data, design_main, "alcohol_use", "Alcohol use")

drug_block <- make_weighted_block(analysis_data, design_main, "drug_use", "Drug use")

# Total row

table1_total <- tibble(

Variable = "Total",

Category = "",

n = nrow(analysis_data),

Percent = 100.0,

Smokers_Percent = NA_real_

)

# Combine all

table1_data <- bind_rows(

table1_total,

smoking_block,

sex_block,

age_block,

mig_block,

marital_block,

edu_block,

emp_block,

res_block,

lang_block,

alc_block,

drug_block

)

# Make category display prettier

table1_data <- table1_data %>%

mutate(

`% of current smokers` = ifelse(is.na(Smokers_Percent), "", sprintf("%.1f", Smokers_Percent)),

`%` = sprintf("%.1f", Percent)

)

# Print table

gt_table1 <- gt(table1_data, groupname_col = "Variable") %>%

tab_header(

title = paste0("Table 1: Overview of study population characteristics (n=", scales::number(nrow(analysis_data), big.mark = "'"), ")")

) %>%

cols_label(

Category = "Variable",

n = "n",

`%` = "%",

`% of current smokers` = "% of current smokers"

) %>%

fmt_number(columns = n, decimals = 0, use_seps = TRUE, sep_mark = "'") %>%

tab_style(

style = cell_text(weight = "bold"),

locations = cells_row_groups()

) %>%

tab_source_note(

source_note = md("Note: *including registered partnerships; **Outside of labour force, referring to retirees. Percentages are survey-weighted; counts are unweighted.")

)

gt_table1

# Export to Word

gtsave(gt_table1, "Table1_weighted.docx")

# Mann-Whitney-U and Chi-squared test:

# --- Mark Included vs Excluded for the main model (unchanged) ---

model_vars <- c("smoker_binary","sex","alter7","maritalstatus_recoded",

"statmigr","AUSBILD3","erwerb","stala","sprache",

"alcohol_use","drug_use")

merged_data <- merged_data %>%

dplyr::mutate(

included = ifelse(complete.cases(dplyr::across(dplyr::all_of(model_vars))),

"Included", "Excluded")

)

# --- Wilcoxon tests you already ran (keep as-is) ---

# w_age, w_edu, w_alc, w_drug and their summaries ...

# --- Safe chi-square helper (unchanged) ---

chisq_safe <- function(x, grp, B = 10000) {

tbl <- table(x, grp, useNA = "no")

suppressWarnings({

test0 <- chisq.test(tbl, correct = FALSE)

})

exp_ok <- all(test0$expected >= 5)

if (!exp_ok) {

test <- chisq.test(tbl, simulate.p.value = TRUE, B = B)

method <- paste0("Chi-square (Monte Carlo, B=", B, ")")

} else {

test <- test0

method <- "Pearson Chi-square"

}

tibble::tibble(p.value = test$p.value, method = method)

}

# --- Chi-square variables (ADD maritalstatus_recoded; optionally include smoker_binary) ---

vars_nominal <- c(

"sex",

"maritalstatus_recoded", # <-- added

"statmigr",

"stala",

"sprache",

"erwerb",

"smoker_binary"

)

chi_out <- lapply(vars_nominal, function(v) {

out <- chisq_safe(merged_data[[v]], merged_data$included)

tibble::tibble(

Variable = v,

Test = out$method,

`P-value` = ifelse(out$p.value < 0.001, "<0.001",

sprintf("%.3f", out$p.value))

)

}) %>% dplyr::bind_rows()

cat("\nNominal-variable tests (Included vs Excluded):\n")

print(chi_out)

## ------------------------------------------------------------

## Add effect sizes for Wilcoxon and Chi-square tests

## Requires: rstatix (for Wilcoxon r) and effectsize (for Cramér's V)

## ------------------------------------------------------------

# install.packages(c("rstatix","effectsize"))

install.packages("rstatix")

install.packages("effectsize")

install.packages("coin")

library(dplyr)

library(tibble)

library(rstatix) # wilcox_effsize

library(effectsize) # cramers_v

## ---------- WILCOXON (Mann–Whitney) with effect size r ----------

# Age (numeric)

w_age <- wilcox.test(alter ~ included, data = merged_data, exact = FALSE)

eff_age <- merged_data %>%

filter(!is.na(alter), !is.na(included)) %>%

wilcox_effsize(alter ~ included, ci = TRUE)

# Education score (ordered 1<2<3)

edu_scores <- merged_data %>%

transmute(included,

AUSBILD3_num = dplyr::case_when(

AUSBILD3 == "Compulsory school or less" ~ 1,

AUSBILD3 == "Secondary" ~ 2,

AUSBILD3 == "Tertiary" ~ 3,

TRUE ~ NA_real_

))

w_edu <- wilcox.test(AUSBILD3_num ~ included, data = edu_scores, exact = FALSE)

eff_edu <- edu_scores %>%

filter(!is.na(AUSBILD3_num), !is.na(included)) %>%

wilcox_effsize(AUSBILD3_num ~ included, ci = TRUE)

# Alcohol frequency code (TALKO15: 1=daily … 8=never)

w_alc <- wilcox.test(TALKO15 ~ included, data = merged_data, exact = FALSE)

eff_alc <- merged_data %>%

filter(!is.na(TALKO15), !is.na(included)) %>%

wilcox_effsize(TALKO15 ~ included, ci = TRUE)

# Drug use recency score (1..4)

drug_scores <- merged_data %>%

transmute(included,

drug_ord = dplyr::case_when(

drogcons == 1 ~ 1, # Never

drogcons == 3 ~ 2, # >12 months ago

drogcons == 4 ~ 3, # past 12 months

drogcons == 5 ~ 4, # past 30 days

TRUE ~ NA_real_

))

w_drug <- wilcox.test(drug_ord ~ included, data = drug_scores, exact = FALSE)

eff_drug <- drug_scores %>%

filter(!is.na(drug_ord), !is.na(included)) %>%

wilcox_effsize(drug_ord ~ included, ci = TRUE)

# Collect Wilcoxon results + effect sizes

wilcox_out <- bind_rows(

tibble(Variable = "Age (years)",

Test = "Mann–Whitney U / Wilcoxon rank-sum",

`P-value` = ifelse(w_age$p.value < 0.001, "<0.001", sprintf("%.3f", w_age$p.value)),

Effect = sprintf("r = %.3f [%0.3f, %0.3f]", eff_age$effsize, eff_age$conf.low, eff_age$conf.high)),

tibble(Variable = "Education (ordered score 1<2<3)",

Test = "Mann–Whitney U / Wilcoxon rank-sum",

`P-value` = ifelse(w_edu$p.value < 0.001, "<0.001", sprintf("%.3f", w_edu$p.value)),

Effect = sprintf("r = %.3f [%0.3f, %0.3f]", eff_edu$effsize, eff_edu$conf.low, eff_edu$conf.high)),

tibble(Variable = "Alcohol frequency code (1=daily … 8=never)",

Test = "Mann–Whitney U / Wilcoxon rank-sum",

`P-value` = ifelse(w_alc$p.value < 0.001, "<0.001", sprintf("%.3f", w_alc$p.value)),

Effect = sprintf("r = %.3f [%0.3f, %0.3f]", eff_alc$effsize, eff_alc$conf.low, eff_alc$conf.high)),

tibble(Variable = "Drug use recency (ordered score 1..4)",

Test = "Mann–Whitney U / Wilcoxon rank-sum",

`P-value` = ifelse(w_drug$p.value < 0.001, "<0.001", sprintf("%.3f", w_drug$p.value)),

Effect = sprintf("r = %.3f [%0.3f, %0.3f]", eff_drug$effsize, eff_drug$conf.low, eff_drug$conf.high))

)

## ---------- CHI-SQUARE with Cramér’s V (bias-corrected) ----------

chisq_safe <- function(x, grp, B = 10000) {

tbl <- table(x, grp, useNA = "no")

suppressWarnings({

test0 <- chisq.test(tbl, correct = FALSE)

})

exp_ok <- all(test0$expected >= 5)

if (!exp_ok) {

test <- chisq.test(tbl, simulate.p.value = TRUE, B = B)

method <- paste0("Chi-square (Monte Carlo, B=", B, ")")

} else {

test <- test0

method <- "Pearson Chi-square"

}

list(test = test, method = method, table = tbl)

}

cramer_with_ci <- function(tbl) {

# bias-corrected Cramér's V with 95% CI

out <- effectsize::cramers_v(tbl, ci = 0.95, bias.correct = TRUE)

# returns a data.frame with Cramers_v, CI_low, CI_high, df, n_Obs

out

}

vars_nominal <- c(

"sex",

"maritalstatus_recoded",

"statmigr",

"stala",

"sprache",

"erwerb",

"smoker_binary"

)

chi_list <- lapply(vars_nominal, function(v) {

res <- chisq_safe(merged_data[[v]], merged_data$included)

v_eff <- cramer_with_ci(res$table)

tibble(

Variable = dplyr::case_when(

v == "sex" ~ "Sex",

v == "maritalstatus_recoded" ~ "Marital status",

v == "statmigr" ~ "Migration background",

v == "stala" ~ "Residence",

v == "sprache" ~ "Language region",

v == "erwerb" ~ "Employment status",

v == "smoker_binary" ~ "Smoking status",

TRUE ~ v

),

Test = res$method,

`P-value`= ifelse(res$test$p.value < 0.001, "<0.001", sprintf("%.3f", res$test$p.value)),

Effect = sprintf("Cramér's V = %.3f [%0.3f, %0.3f]",

v_eff$Cramers_v, v_eff$CI_low, v_eff$CI_high)

)

})

chi_out <- bind_rows(chi_list)

## ---------- Combined test summary (Wilcoxon + Chi-square) ----------

test_results <- bind_rows(wilcox_out, chi_out) %>%

# optional: order to match your reporting order

mutate(Variable = factor(Variable, levels = c(

"Sex",

"Age (years)",

"Marital status",

"Migration background",

"Education (ordered score 1<2<3)",

"Employment status",

"Residence",

"Language region",

"Alcohol frequency code (1=daily … 8=never)",

"Drug use recency (ordered score 1..4)",

"Smoking status"

))) %>%

arrange(Variable)

print(test_results)

Step 2:

# EFFECT MODIFICATION (INTERACTION) TESTS — WEIGHTED VERSION

# ---- 0) Ensure intended reference levels BEFORE fitting models ----

merged_data <- merged_data %>%

mutate(

sex = fct_relevel(sex, "Male"),

alter7 = fct_relevel(alter7, "15–24"),

maritalstatus_recoded = fct_relevel(maritalstatus_recoded, "Married/Registered Partnership"),

statmigr = fct_relevel(statmigr, "No migration background"),

AUSBILD3 = fct_relevel(AUSBILD3, "Compulsory school or less"),

erwerb = fct_relevel(erwerb, "Employed"),

stala = fct_relevel(stala, "Urban"),

sprache = fct_relevel(sprache, "German"),

alcohol_use = fct_relevel(alcohol_use, "Abstinent"),

drug_use = fct_relevel(drug_use, "Never")

)

# ---- 1) Ensure numeric smoking outcome exists for svyglm ----

# If you already created smoker_binary_num earlier, this will simply keep it.

if (!"smoker_binary_num" %in% names(merged_data)) {

merged_data <- merged_data %>%

mutate(

smoker_binary_num = case_when(

smoker_binary == "Smoker" ~ 1,

smoker_binary == "Non-smoker" ~ 0,

TRUE ~ NA_real_

)

)

}

# ---- 2) Define the variables that determine inclusion in the weighted main model ----

model_vars_w <- c(

"smoker_binary_num",

"sex",

"alter7",

"maritalstatus_recoded",

"statmigr",

"AUSBILD3",

"erwerb",

"stala",

"sprache",

"alcohol_use",

"drug_use",

"wght"

)

# Keep the exact complete-case dataset used by the weighted interaction models

model_data_w <- merged_data %>%

filter(complete.cases(across(all_of(model_vars_w)))) %>%

droplevels()

# Sanity check N

N_model_data_w <- nrow(model_data_w)

cat("\nN in complete-case weighted model dataset =", N_model_data_w, "\n")

# ---- 3) Create survey design object ----

design_main_w <- svydesign(

ids = ~1,

weights = ~wght,

data = model_data_w

)

# ---- 4) Fit weighted main-effects (baseline) model ----

model_main_w <- svyglm(

smoker_binary_num ~ statmigr + sex + alter7 + maritalstatus_recoded +

AUSBILD3 + erwerb + stala + sprache + alcohol_use + drug_use,

design = design_main_w,

family = quasibinomial()

)

cat("Weighted main model complete-case N =", nrow(model_data_w), "\n")

# ---- 5) Fit weighted interaction models (ONE interaction at a time) ----

# 5a) statmigr × sex

model_int_sex_w <- svyglm(

smoker_binary_num ~ statmigr * sex + alter7 + maritalstatus_recoded +

AUSBILD3 + erwerb + stala + sprache + alcohol_use + drug_use,

design = design_main_w,

family = quasibinomial()

)

# 5b) statmigr × age group (alter7)

model_int_age_w <- svyglm(

smoker_binary_num ~ statmigr * alter7 + sex + maritalstatus_recoded +

AUSBILD3 + erwerb + stala + sprache + alcohol_use + drug_use,

design = design_main_w,

family = quasibinomial()

)

# 5c) statmigr × education (AUSBILD3)

model_int_edu_w <- svyglm(

smoker_binary_num ~ statmigr * AUSBILD3 + sex + alter7 + maritalstatus_recoded +

erwerb + stala + sprache + alcohol_use + drug_use,

design = design_main_w,

family = quasibinomial()

)

# ---- 6) Design-based Wald tests for interaction terms ----

# This replaces the old likelihood ratio tests.

wald_sex <- regTermTest(model_int_sex_w, ~ statmigr:sex)

wald_age <- regTermTest(model_int_age_w, ~ statmigr:alter7)

wald_edu <- regTermTest(model_int_edu_w, ~ statmigr:AUSBILD3)

cat("\n--- Design-based Wald test: statmigr × sex ---\n")

print(wald_sex)

cat("\n--- Design-based Wald test: statmigr × alter7 ---\n")

print(wald_age)

cat("\n--- Design-based Wald test: statmigr × AUSBILD3 ---\n")

print(wald_edu)

# ---- 7) Create a compact manuscript-friendly summary table of interaction tests ----

extract_wald <- function(wald_obj, label, N_value) {

# regTermTest objects usually provide:

# - Ftest

# - df

# - p

#

# We extract as robustly as possible.

p_val <- tryCatch(as.numeric(wald_obj$p), error = function(e) NA_real_)

F_val <- tryCatch(as.numeric(wald_obj$Ftest), error = function(e) NA_real_)

# df may be length 2 (numerator, denominator); convert to a readable string

df_val <- tryCatch({

d <- wald_obj$df

if (length(d) == 2) {

paste0(d[1], ", ", d[2])

} else if (length(d) == 1) {

as.character(d)

} else {

NA_character_

}

}, error = function(e) NA_character_)

tibble(

Interaction = label,

`Wald F` = ifelse(is.na(F_val), NA_character_, sprintf("%.2f", F_val)),

`df` = df_val,

`p-value` = ifelse(is.na(p_val), NA_character_,

ifelse(p_val < 0.001, "<0.001", sprintf("%.3f", p_val))),

N = N_value

)

}

interaction_tests_w <- bind_rows(

extract_wald(wald_sex, "Migration background × Sex", N_model_data_w),

extract_wald(wald_age, "Migration background × Age group", N_model_data_w),

extract_wald(wald_edu, "Migration background × Education", N_model_data_w)

)

cat("\n--- Interaction test summary (design-based Wald tests) ---\n")

print(interaction_tests_w)

# ---- 8) Save the interaction test summary as a Word file ----

gt_interactions_w <- gt(interaction_tests_w) %>%

tab_header(

title = "Design-based Wald tests for effect modification",

subtitle = "Survey-weighted models with interaction terms"

)

gtsave(gt_interactions_w, "Interaction_Wald_summary_weighted.docx")

getwd()

Step 3:

Multivariable logistic regression analysis:

# Keep variables INCLUDING weight

merged_data <- merged_data[, c(

"idno", "wght", "alter", "TABAC3", "sex", "maritalstatus", "statmigr", "stala",

"sprache", "AUSBILD3", "erwerb", "TALKO15", "drogcons"

)]

# -----------------------------

# Step 1: Clean missing values

# -----------------------------

invalid_codes <- c(-1, -2, -3, -4, -5, -6, -8)

vars_to_clean <- c(

"alter", "TABAC3", "sex", "maritalstatus", "statmigr", "stala",

"sprache", "AUSBILD3", "erwerb", "TALKO15", "drogcons"

)

merged_data[vars_to_clean] <- lapply(merged_data[vars_to_clean], function(x) {

x <- as.numeric(as.character(x))

x[x %in% invalid_codes] <- NA

return(x)

})

# Clean weight variable

merged_data$wght <- as.numeric(as.character(merged_data$wght))

merged_data$wght[!is.finite(merged_data$wght) | merged_data$wght <= 0] <- NA

# -----------------------------

# Step 2: Create derived variables

# -----------------------------

merged_data <- merged_data %>%

mutate(

# Age groups

alter7 = case_when(

alter >= 15 & alter <= 24 ~ "15–24",

alter >= 25 & alter <= 34 ~ "25–34",

alter >= 35 & alter <= 44 ~ "35–44",

alter >= 45 & alter <= 54 ~ "45–54",

alter >= 55 & alter <= 64 ~ "55–64",

alter >= 65 & alter <= 74 ~ "65–74",

alter >= 75 ~ "75+",

TRUE ~ NA_character_

),

# Numeric smoking outcome for weighted regression

smoker_binary_num = case_when(

TABAC3 == 3 ~ 1,

TABAC3 %in% c(1, 2) ~ 0,

TRUE ~ NA_real_

),

# Factor smoking outcome for display

smoker_binary = factor(

smoker_binary_num,

levels = c(0, 1),

labels = c("Non-smoker", "Smoker")

),

# Marital status

maritalstatus_recoded = case_when(

maritalstatus %in% c(2, 6) ~ 1,

maritalstatus %in% c(1, 3, 4, 5, 7) ~ 2,

TRUE ~ NA_real_

),

# Alcohol use

alcohol_use = case_when(

TALKO15 == 8 ~ 1,

TALKO15 %in% c(5, 6, 7) ~ 2,

TALKO15 %in% c(1, 2, 3, 4) ~ 3,

TRUE ~ NA_real_

),

# Drug use

drug_use = case_when(

drogcons == 5 ~ 4,

drogcons == 4 ~ 3,

drogcons == 3 ~ 2,

drogcons == 1 ~ 1,

TRUE ~ NA_real_

)

)

# -----------------------------

# Step 3: Convert variables to factors with readable labels

# -----------------------------

merged_data <- merged_data %>%

mutate(

alter7 = factor(alter7, levels = c("15–24", "25–34", "35–44", "45–54", "55–64", "65–74", "75+")),

sex = factor(sex, levels = c(1, 2), labels = c("Male", "Female")),

maritalstatus_recoded = factor(maritalstatus_recoded, levels = c(1, 2),

labels = c("Married/Registered Partnership", "Unmarried")),

statmigr = factor(statmigr, levels = c(1, 2, 3),

labels = c("No migration background", "1st generation", "2nd or higher generation")),

stala = factor(stala, levels = c(1, 2, 3),

labels = c("Urban", "Peri-urban", "Rural")),

sprache = factor(sprache, levels = c(1, 2, 3),

labels = c("German", "French", "Italian")),

erwerb = factor(erwerb, levels = c(3, 1, 2),

labels = c("Employed", "Not working", "Unemployed")),

AUSBILD3 = factor(AUSBILD3, levels = c(1, 2, 3),

labels = c("Compulsory school or less", "Secondary", "Tertiary")),

alcohol_use = factor(alcohol_use, levels = c(1, 2, 3),

labels = c("Abstinent", "Occasional", "Frequent")),

drug_use = factor(drug_use, levels = c(1, 2, 3, 4),

labels = c("Never", "more than 12 months ago", "in the past 12 months", "in the past 30 days"))

)

# -----------------------------

# Set intended reference levels

# -----------------------------

merged_data <- merged_data %>%

mutate(

sex = fct_relevel(sex, "Male"),

alter7 = fct_relevel(alter7, "15–24"),

maritalstatus_recoded = fct_relevel(maritalstatus_recoded, "Married/Registered Partnership"),

statmigr = fct_relevel(statmigr, "No migration background"),

stala = fct_relevel(stala, "Urban"),

sprache = fct_relevel(sprache, "German"),

erwerb = fct_relevel(erwerb, "Employed"),

alcohol_use = fct_relevel(alcohol_use, "Abstinent"),

AUSBILD3 = fct_relevel(AUSBILD3, "Compulsory school or less"),

drug_use = fct_relevel(drug_use, "Never")

)

## ------------------------------------------------------------

# TABLE S1: Weighted multivariable logistic regression

## ------------------------------------------------------------

model_vars <- c(

"smoker_binary_num", "sex", "alter7", "maritalstatus_recoded",

"statmigr", "AUSBILD3", "erwerb", "stala", "sprache",

"alcohol_use", "drug_use", "wght"

)

model_data <- merged_data %>%

filter(complete.cases(across(all_of(model_vars)))) %>%

droplevels()

cat("\nWeighted complete-case N =", nrow(model_data), "\n")

design_main <- svydesign(

ids = ~1,

weights = ~wght,

data = model_data

)

model1_w <- svyglm(

smoker_binary_num ~ sex + alter7 + maritalstatus_recoded +

statmigr + AUSBILD3 + erwerb + stala + sprache + alcohol_use + drug_use,

design = design_main,

family = quasibinomial()

)

summary(model1_w)

# By sex in analytic sample

dplyr::count(model_data, sex, name = "n")

# -----------------------------

# Table S1 mirroring the forest plot

# -----------------------------

tbl <- tidy(model1_w, conf.int = TRUE) %>%

filter(term != "(Intercept)") %>%

mutate(

estimate = exp(estimate),

conf.low = exp(conf.low),

conf.high = exp(conf.high),

term = str_replace_all(term, "`", ""),

term = term %>%

str_replace("^sex", "") %>%

str_replace("^alter7", "") %>%

str_replace("^maritalstatus_recoded", "") %>%

str_replace("^statmigr", "") %>%

str_replace("^stala", "") %>%

str_replace("^sprache", "") %>%

str_replace("^erwerb", "") %>%

str_replace("^alcohol_use", "") %>%

str_replace("^AUSBILD3", "") %>%

str_replace("^drug_use", "") %>%

str_squish(),

term = str_replace_all(term, "–", "-")

)

tbl <- tbl %>%

mutate(

group = case_when(

term %in% c("Male","Female") ~ "Sex",

str_detect(term, "^(15-24|25-34|35-44|45-54|55-64|65-74|75\\+)") ~ "Age",

term %in% c("Married/Registered Partnership","Unmarried") ~ "Marital status",

term %in% c("No migration background","1st generation","2nd or higher generation") ~ "Migration background",

term %in% c("Compulsory school or less","Secondary","Tertiary") ~ "Education",

term %in% c("Employed","Not working","Unemployed") ~ "Employment status",

term %in% c("Urban","Peri-urban","Rural") ~ "Residence",

term %in% c("German","French","Italian") ~ "Language region",

term %in% c("Abstinent","Occasional","Frequent") ~ "Alcohol use",

term %in% c("Never","more than 12 months ago","in the past 12 months","in the past 30 days") ~ "Drug use",

TRUE ~ "Other"

)

)

refs <- tribble(

~group, ~term,

"Sex", "Male",

"Age", "15-24",

"Marital status", "Married/Registered Partnership",

"Migration background","No migration background",

"Education", "Compulsory school or less",

"Employment status", "Employed",

"Residence", "Urban",

"Language region", "German",

"Alcohol use", "Abstinent",

"Drug use", "Never"

) %>%

mutate(

OR = "1 (ref)", `95% CI` = "", `P-value` = ""

)

group_order <- c(

"Sex","Age","Marital status","Migration background","Education",

"Employment status","Residence","Language region","Alcohol use","Drug use"

)

desired_levels <- tribble(

~group, ~term, ~ord,

"Sex", "Male", 1,

"Sex", "Female", 2,

"Age", "15-24", 1,

"Age", "25-34", 2,

"Age", "35-44", 3,

"Age", "45-54", 4,

"Age", "55-64", 5,

"Age", "65-74", 6,

"Age", "75+", 7,

"Marital status", "Married/Registered Partnership", 1,

"Marital status", "Unmarried", 2,

"Migration background","No migration background", 1,

"Migration background","1st generation", 2,

"Migration background","2nd or higher generation", 3,

"Education", "Compulsory school or less", 1,

"Education", "Secondary", 2,

"Education", "Tertiary", 3,

"Employment status", "Employed", 1,

"Employment status", "Not working", 2,

"Employment status", "Unemployed", 3,

"Residence", "Urban", 1,

"Residence", "Peri-urban", 2,

"Residence", "Rural", 3,

"Language region", "German", 1,

"Language region", "French", 2,

"Language region", "Italian", 3,

"Alcohol use", "Abstinent", 1,

"Alcohol use", "Occasional", 2,

"Alcohol use", "Frequent", 3,

"Drug use", "Never", 1,

"Drug use", "more than 12 months ago", 2,

"Drug use", "in the past 12 months", 3,

"Drug use", "in the past 30 days", 4

)

ordered_tbl <- tbl %>%

mutate(group = factor(group, levels = group_order)) %>%

left_join(desired_levels, by = c("group","term"))

missing_map <- ordered_tbl %>% filter(is.na(ord)) %>% distinct(group, term)

if (nrow(missing_map) > 0) {

message("Terms not found in desired_levels (check spelling/dashes):")

print(missing_map)

}

ordered_tbl <- ordered_tbl %>%

mutate(

term = factor(

term,

levels = desired_levels %>%

arrange(match(group, group_order), ord) %>%

pull(term) %>%

unique()

),

OR_num = estimate,

OR = sprintf("%.2f", estimate),

`95% CI` = paste0("[", sprintf("%.2f", conf.low), ", ", sprintf("%.2f", conf.high), "]"),

`P-value` = ifelse(p.value < 0.001, "<0.001", sprintf("%.3f", p.value))

) %>%

select(group, term, OR, `95% CI`, `P-value`, OR_num, ord)

final_table <- refs %>%

bind_rows(

ordered_tbl %>%

arrange(match(group, group_order), ord)

)

gt_tbl <- gt(final_table, groupname_col = "group") %>%

tab_header(

title = "Adjusted Odds Ratios for Current Smoking",

subtitle = "Survey-weighted multivariable logistic regression model"

) %>%

cols_label(

term = "Category",

OR = "Odds Ratio",

`95% CI` = "95% Confidence Interval",

`P-value` = "P-value"

) %>%

cols_hide(columns = c(OR_num, ord)) %>%

tab_style(

style = cell_text(weight = "bold"),

locations = cells_row_groups(groups = group_order)

)

gt_tbl

gtsave(gt_tbl, "SM3_Table_S1_weighted.docx")

## ------------------------------------------------------------

# FIGURE S1: Weighted forest plot

## ------------------------------------------------------------

clean_term <- function(x) {

x %>%

stringr::str_replace_all("`", "") %>%

stringr::str_replace("^sex", "") %>%

stringr::str_replace("^alter7", "") %>%

stringr::str_replace("^maritalstatus_recoded", "") %>%

stringr::str_replace("^statmigr", "") %>%

stringr::str_replace("^stala", "") %>%

stringr::str_replace("^sprache", "") %>%

stringr::str_replace("^erwerb", "") %>%

stringr::str_replace("^alcohol_use", "") %>%

stringr::str_replace("^AUSBILD3", "") %>%

stringr::str_replace("^drug_use", "") %>%

stringr::str_squish()

}

plot_data <- broom::tidy(model1_w, conf.int = TRUE) %>%

dplyr::filter(term != "(Intercept)") %>%

dplyr::mutate(

estimate = exp(estimate),

conf.low = exp(conf.low),

conf.high = exp(conf.high),

term = clean_term(term),

reference = FALSE

)

reference_rows <- tibble::tribble(

~term, ~estimate, ~conf.low, ~conf.high, ~reference,

"Male", 1, NA, NA, TRUE,

"15–24", 1, NA, NA, TRUE,

"Married/Registered Partnership", 1, NA, NA, TRUE,

"No migration background", 1, NA, NA, TRUE,

"Compulsory school or less", 1, NA, NA, TRUE,

"Employed", 1, NA, NA, TRUE,

"Urban", 1, NA, NA, TRUE,

"German", 1, NA, NA, TRUE,

"Abstinent", 1, NA, NA, TRUE,

"Never", 1, NA, NA, TRUE

)

plot_data <- dplyr::bind_rows(plot_data, reference_rows)

plot_data <- plot_data %>%

mutate(

group = case_when(

term %in% c("Male","Female") ~ "Sex",

str_detect(term, "^(15–24|25–34|35–44|45–54|55–64|65–74|75\\+)") ~ "Age",

term %in% c("Married/Registered Partnership","Unmarried") ~ "Marital status",

term %in% c("No migration background","1st generation","2nd or higher generation") ~ "Migration background",

term %in% c("Compulsory school or less","Secondary","Tertiary") ~ "Education",

term %in% c("Employed","Not working","Unemployed") ~ "Employment status",

term %in% c("Urban","Peri-urban","Rural") ~ "Residence",

term %in% c("German","French","Italian") ~ "Language region",

term %in% c("Abstinent","Occasional","Frequent") ~ "Alcohol use",

term %in% c("Never","more than 12 months ago","in the past 12 months","in the past 30 days") ~ "Drug use",

TRUE ~ "Other"

)

)

group_order <- c(

"Sex","Age","Marital status","Migration background","Education",

"Employment status","Residence","Language region","Alcohol use","Drug use"

)

plot_data <- plot_data %>%

mutate(group = factor(group, levels = group_order)) %>%

mutate(term = str_replace_all(term, "–", "-"))

desired_levels <- tibble::tribble(

~group, ~term, ~ord,

"Sex", "Male", 1,

"Sex", "Female", 2,

"Age", "15-24", 1,

"Age", "25-34", 2,

"Age", "35-44", 3,

"Age", "45-54", 4,

"Age", "55-64", 5,

"Age", "65-74", 6,

"Age", "75+", 7,

"Marital status", "Married/Registered Partnership", 1,

"Marital status", "Unmarried", 2,

"Migration background","No migration background", 1,

"Migration background","1st generation", 2,

"Migration background","2nd or higher generation", 3,

"Education", "Compulsory school or less", 1,

"Education", "Secondary", 2,

"Education", "Tertiary", 3,

"Employment status", "Employed", 1,

"Employment status", "Not working", 2,

"Employment status", "Unemployed", 3,

"Residence", "Urban", 1,

"Residence", "Peri-urban", 2,

"Residence", "Rural", 3,

"Language region", "German", 1,

"Language region", "French", 2,

"Language region", "Italian", 3,

"Alcohol use", "Abstinent", 1,

"Alcohol use", "Occasional", 2,

"Alcohol use", "Frequent", 3,

"Drug use", "Never", 1,

"Drug use", "more than 12 months ago", 2,

"Drug use", "in the past 12 months", 3,

"Drug use", "in the past 30 days", 4

)

desired_levels <- desired_levels %>%

mutate(group = factor(group, levels = group_order))

plot_data <- plot_data %>%

left_join(desired_levels, by = c("group","term")) %>%

mutate(group = factor(group, levels = group_order))

missing_map <- plot_data %>% filter(is.na(ord)) %>% distinct(group, term)

if (nrow(missing_map) > 0) {

message("Terms not found in desired_levels (check dashes/spelling):")

print(missing_map)

}

term_levels <- desired_levels %>%

arrange(match(as.character(group), group_order), ord) %>%

pull(term) %>%

unique()

plot_data <- plot_data %>%

mutate(term = factor(term, levels = term_levels))

range_vals <- plot_data %>% filter(!reference, is.finite(conf.low), is.finite(conf.high))

xmin <- if (nrow(range_vals)) min(range_vals$conf.low, na.rm = TRUE) else 0.5

xmax <- if (nrow(range_vals)) max(range_vals$conf.high, na.rm = TRUE) else 2

xmin <- min(xmin, 0.5)

xmax <- xmax * 1.1

xmin <- min(xmin, 1 / 1.5)

xmax <- max(xmax, 1.5)

candidate_breaks <- c(0.25, 0.5, 1, 2, 3, 4, 5, 8, 10, 15, 20, 30)

breaks_use <- candidate_breaks[candidate_breaks >= xmin & candidate_breaks <= xmax]

if (!length(breaks_use)) breaks_use <- c(0.5, 1, 2, 5, 10)

p <- ggplot(plot_data, aes(x = estimate, y = term)) +

geom_vline(xintercept = 1, linetype = "dashed") +

geom_errorbarh(aes(xmin = conf.low, xmax = conf.high),

height = 0.18, linewidth = 0.5, na.rm = TRUE) +

geom_point(aes(shape = reference), size = 2.6, stroke = 0.8, na.rm = TRUE) +

scale_shape_manual(values = c(`FALSE` = 16, `TRUE` = 4),

labels = c("Estimate","Reference")) +

scale_x_log10(breaks = breaks_use, labels = breaks_use, limits = c(xmin, xmax)) +

facet_grid(group ~ ., scales = "free_y", space = "free_y", switch = "y") +

labs(

title = "Adjusted Odds Ratios for Smoking",

x = "Odds Ratio (log scale)",

y = NULL,

shape = NULL

) +

theme_minimal(base_size = 11) +

theme(

legend.position = "right",

axis.text.y = element_text(size = 10),

strip.placement = "outside",

strip.text.y.left = element_text(angle = 0, hjust = 1, face = "bold"),

plot.title = element_text(face = "bold", hjust = 0.5),

panel.grid.minor = element_blank()

)

print(p)

ggsave("SM3_Figure_S1_weighted.png", p, width = 7, height = 10, dpi = 600)

ggsave("SM3_Figure_S1_weighted.pdf", p, width = 7, height = 10)

## ------------------------------------------------------------

# TABLE S2: 1st-generation vs 2nd-or-higher-generation migrants

## ------------------------------------------------------------

mig_compare_data <- model_data %>%

filter(statmigr %in% c("1st generation", "2nd or higher generation")) %>%

droplevels()

# Set reference category to second generation

mig_compare_data <- mig_compare_data %>%

mutate(

statmigr = fct_relevel(statmigr, "2nd or higher generation", "1st generation")

)

design_mig_compare <- svydesign(

ids = ~1,

weights = ~wght,

data = mig_compare_data

)

model_mig_compare_w <- svyglm(

smoker_binary_num ~ statmigr + sex + alter7 + maritalstatus_recoded +

AUSBILD3 + erwerb + stala + sprache + alcohol_use + drug_use,

design = design_mig_compare,

family = quasibinomial()

)

summary(model_mig_compare_w)

mig_compare_tbl <- tidy(model_mig_compare_w, conf.int = TRUE) %>%

filter(str_detect(term, "^statmigr")) %>%

mutate(

OR = exp(estimate),

CI_low = exp(conf.low),

CI_high = exp(conf.high),

Comparison = "First-generation vs. second-generation migrants",

`Adjusted odds ratio (OR)` = sprintf("%.2f", OR),

`95% confidence interval` = paste0(sprintf("%.2f", CI_low), "–", sprintf("%.2f", CI_high)),

`p-value` = ifelse(p.value < 0.001, "<0.001", sprintf("%.3f", p.value))

) %>%

select(

`Migration background comparison` = Comparison,

`Adjusted odds ratio (OR)`,

`95% confidence interval`,

`p-value`

)

gt_mig_compare <- gt(mig_compare_tbl) %>%

tab_header(

title = "Adjusted comparison between first- and second-generation migrants",

subtitle = "Survey-weighted multivariable logistic regression"

)

gt_mig_compare

gtsave(gt_mig_compare, "SM3_Table_S2_weighted.docx")

## ------------------------------------------------------------

# WEIGHTED OVERVIEW OF SMOKING IN THE ANALYTIC SAMPLE

## ------------------------------------------------------------

smoking_prev <- svymean(~smoker_binary_num, design_main, na.rm = TRUE)

print(smoking_prev)

smoking_overview_model <- tibble(

Smoking_Status = c("Non-smoker", "Smoker"),

Weighted_Proportion = c(

1 - coef(smoking_prev)[1],

coef(smoking_prev)[1]

),

Percent = round(100 * Weighted_Proportion, 1)

)

print(smoking_overview_model)

# Optional raw counts in analytic sample

smoking_counts_model <- model_data %>%

count(smoker_binary) %>%

mutate(

Total = sum(n),

Percent_unweighted = round(100 * n / Total, 1)

) %>%

select(Smoking_Status = smoker_binary, N = n, Percent_unweighted)

print(smoking_counts_model)

# FIND N in ANALYSIS 1

nobs(model1)

# 19441

# By sex

mf <- model.frame(model1) # the data actually used

dplyr::count(mf, sex, name = "n")

#Output # 1 Male 8999, 2 Female 10442

Step 4:

Stratified Analyses

Stratified analysis 1: sex

# Keep variables INCLUDING weight

merged_data <- merged_data[, c(

"idno", "wght", "alter", "TABAC3", "sex", "maritalstatus", "statmigr", "stala",

"sprache", "AUSBILD3", "erwerb", "TALKO15", "drogcons"

)]

# -----------------------------

# Step 1: Clean missing values

# -----------------------------

invalid_codes <- c(-1, -2, -3, -4, -5, -6, -8)

vars_to_clean <- c(

"alter", "TABAC3", "sex", "maritalstatus", "statmigr", "stala",

"sprache", "AUSBILD3", "erwerb", "TALKO15", "drogcons"

)

merged_data[vars_to_clean] <- lapply(merged_data[vars_to_clean], function(x) {

x <- as.numeric(as.character(x))

x[x %in% invalid_codes] <- NA

return(x)

})

# Clean weight variable

merged_data$wght <- as.numeric(as.character(merged_data$wght))

merged_data$wght[!is.finite(merged_data$wght) | merged_data$wght <= 0] <- NA

# -----------------------------

# Step 2: Create derived variables

# -----------------------------

merged_data <- merged_data %>%

mutate(

# Age groups

alter7 = case_when(

alter >= 15 & alter <= 24 ~ "15–24",

alter >= 25 & alter <= 34 ~ "25–34",

alter >= 35 & alter <= 44 ~ "35–44",

alter >= 45 & alter <= 54 ~ "45–54",

alter >= 55 & alter <= 64 ~ "55–64",

alter >= 65 & alter <= 74 ~ "65–74",

alter >= 75 ~ "75+",

TRUE ~ NA_character_

),

# Numeric smoking outcome for weighted logistic regression

smoker_binary_num = case_when(

TABAC3 == 3 ~ 1,

TABAC3 %in% c(1, 2) ~ 0,

TRUE ~ NA_real_

),

# Factor smoking outcome for tables / counts

smoker_binary = factor(

smoker_binary_num,

levels = c(0, 1),

labels = c("Non-smoker", "Smoker")

),

# Marital status: 1 = Married/Registered, 2 = Unmarried

maritalstatus_recoded = case_when(

maritalstatus %in% c(2, 6) ~ 1,

maritalstatus %in% c(1, 3, 4, 5, 7) ~ 2,

TRUE ~ NA_real_

),

# Alcohol use: 3 = Frequent, 2 = Occasional, 1 = Abstinent

alcohol_use = case_when(

TALKO15 == 8 ~ 1,

TALKO15 %in% c(5, 6, 7) ~ 2,

TALKO15 %in% c(1, 2, 3, 4) ~ 3,

TRUE ~ NA_real_

),

# Drug use: 4 = in the past 30 days, 3 = in the past 12 months,

# 2 = more than 12 months ago, 1 = Never consumed

drug_use = case_when(

drogcons == 5 ~ 4,

drogcons == 4 ~ 3,

drogcons == 3 ~ 2,

drogcons == 1 ~ 1,

TRUE ~ NA_real_

)

)

# -----------------------------

# Step 3: Convert variables to factors with readable labels

# -----------------------------

merged_data <- merged_data %>%

mutate(

alter7 = factor(alter7, levels = c("15–24", "25–34", "35–44", "45–54", "55–64", "65–74", "75+")),

sex = factor(sex, levels = c(1, 2), labels = c("Male", "Female")),

maritalstatus_recoded = factor(maritalstatus_recoded, levels = c(1, 2),

labels = c("Married/Registered Partnership", "Unmarried")),

statmigr = factor(statmigr, levels = c(1, 2, 3),

labels = c("No migration background", "1st generation", "2nd or higher generation")),

stala = factor(stala, levels = c(1, 2, 3),

labels = c("Urban", "Peri-urban", "Rural")),

sprache = factor(sprache, levels = c(1, 2, 3),

labels = c("German", "French", "Italian")),

erwerb = factor(erwerb, levels = c(3, 1, 2),

labels = c("Employed", "Not working", "Unemployed")),

AUSBILD3 = factor(AUSBILD3, levels = c(1, 2, 3),

labels = c("Compulsory school or less", "Secondary", "Tertiary")),

alcohol_use = factor(alcohol_use, levels = c(1, 2, 3),

labels = c("Abstinent", "Occasional", "Frequent")),

drug_use = factor(drug_use, levels = c(1, 2, 3, 4),

labels = c("Never", "more than 12 months ago", "in the past 12 months", "in the past 30 days"))

)

# -----------------------------

# Keep reference levels consistent

# -----------------------------

merged_data <- merged_data %>%

mutate(

sex = fct_relevel(sex, "Male"),

alter7 = fct_relevel(alter7, "15–24","25–34","35–44","45–54","55–64","65–74","75+"),

maritalstatus_recoded = fct_relevel(maritalstatus_recoded, "Married/Registered Partnership","Unmarried"),

statmigr = fct_relevel(statmigr, "No migration background", "1st generation", "2nd or higher generation"),

AUSBILD3 = fct_relevel(AUSBILD3, "Compulsory school or less","Secondary","Tertiary"),

erwerb = fct_relevel(erwerb, "Employed","Not working","Unemployed"),

stala = fct_relevel(stala, "Urban","Peri-urban","Rural"),

sprache = fct_relevel(sprache, "German","French","Italian"),

alcohol_use = fct_relevel(alcohol_use, "Abstinent","Occasional","Frequent"),

drug_use = fct_relevel(drug_use, "Never","more than 12 months ago","in the past 12 months","in the past 30 days")

)

## ------------------------------------------------------------

# MAIN WEIGHTED MODEL

## ------------------------------------------------------------

main_vars <- c(

"smoker_binary_num", "sex", "alter7", "maritalstatus_recoded",

"statmigr", "AUSBILD3", "erwerb", "stala", "sprache",

"alcohol_use", "drug_use", "wght"

)

analysis_data_main <- merged_data %>%

filter(complete.cases(across(all_of(main_vars))))

design_main <- svydesign(

ids = ~1,

weights = ~wght,

data = analysis_data_main

)

model1_w <- svyglm(

smoker_binary_num ~ sex + alter7 + maritalstatus_recoded +

statmigr + AUSBILD3 + erwerb + stala + sprache + alcohol_use + drug_use,

design = design_main,

family = quasibinomial()

)

summary(model1_w)

## ------------------------------------------------------------

# HELPERS FOR STRATIFIED WEIGHTED MODELS

## ------------------------------------------------------------

drop_single_level_covars <- function(dat, covars) {

keep <- covars[

vapply(covars, function(v) {

x <- dat[[v]]

sum(!is.na(x)) > 0 && dplyr::n_distinct(x, na.rm = TRUE) >= 2

}, logical(1))

]

dropped <- setdiff(covars, keep)

if (length(dropped) > 0) {

message("Dropped (single level) in this subset: ", paste(dropped, collapse = ", "))

}

keep

}

tidy_migration_weighted <- function(model, strat_label, strat_name) {

broom::tidy(model, conf.int = TRUE) %>%

filter(term != "(Intercept)", str_detect(term, "^`?statmigr")) %>%

mutate(

term = str_replace_all(term, "`", ""),

term = str_replace(term, "^statmigr", ""),

term = str_squish(term),

OR = exp(estimate),

CI_low = exp(conf.low),

CI_high = exp(conf.high)

) %>%

mutate(term = factor(term, levels = c("1st generation","2nd or higher generation"))) %>%

mutate(!!strat_name := strat_label)

}

## ------------------------------------------------------------

# STRATIFIED ANALYSIS 1: SEX

## ------------------------------------------------------------

# Covariates mirroring main model, minus sex

sex_covars <- c(

"statmigr","alter7","maritalstatus_recoded","AUSBILD3",

"erwerb","stala","sprache","alcohol_use","drug_use"

)

fit_strat_sex <- function(data, sex_level, covars) {

ds <- data %>%

filter(sex == sex_level) %>%

droplevels()

covars_kept <- drop_single_level_covars(ds, covars)

needed_vars <- c("smoker_binary_num", covars_kept, "wght")

ds <- ds %>%

filter(complete.cases(across(all_of(needed_vars))))

design_sub <- svydesign(

ids = ~1,

weights = ~wght,

data = ds

)

fml <- reformulate(covars_kept, response = "smoker_binary_num")

fit <- svyglm(

fml,

design = design_sub,

family = quasibinomial()

)

list(

model = fit,

N = nrow(ds),

Events = sum(ds$smoker_binary_num == 1, na.rm = TRUE),

Non_events = sum(ds$smoker_binary_num == 0, na.rm = TRUE),

Kept_covariates = covars_kept

)

}

fit_male <- fit_strat_sex(merged_data, "Male", sex_covars)

fit_female <- fit_strat_sex(merged_data, "Female", sex_covars)

model_male_w <- fit_male$model

model_female_w <- fit_female$model

effects_male <- tidy_migration_weighted(model_male_w, "Male", "Sex")

effects_female <- tidy_migration_weighted(model_female_w, "Female", "Sex")

migration_effects_sex <- bind_rows(effects_male, effects_female) %>%

arrange(Sex, term)

# --- Plot

rng <- migration_effects_sex %>% filter(is.finite(CI_low), is.finite(CI_high))

xmin <- if (nrow(rng)) min(rng$CI_low) else 0.5

xmax <- if (nrow(rng)) max(rng$CI_high) else 2

xmin <- min(xmin, 0.5)

xmax <- max(xmax * 1.1, 1.5)

breaks_use <- c(0.25, 0.5, 1, 2, 3, 5, 10, 20)

breaks_use <- breaks_use[breaks_use >= xmin & breaks_use <= xmax]

p_mig_sex_w <- ggplot(migration_effects_sex, aes(x = OR, y = term, color = Sex)) +

geom_vline(xintercept = 1, linetype = "dashed") +

geom_errorbarh(aes(xmin = CI_low, xmax = CI_high),

position = position_dodge(width = 0.5),

height = 0.2, linewidth = 0.5) +

geom_point(position = position_dodge(width = 0.5), size = 2.6) +

scale_x_log10(breaks = breaks_use, limits = c(xmin, xmax)) +

labs(

title = "Migration background and current smoking, stratified by sex",

subtitle = "Reference within each sex group: No migration background",

x = "Adjusted Odds Ratio (log scale)",

y = NULL

) +

theme_minimal(base_size = 11) +

theme(

legend.position = "right",

axis.text.y = element_text(size = 10),

plot.title = element_text(face = "bold", hjust = 0.5)

)

print(p_mig_sex_w)

ggsave("stratified_migration_by_sex_weighted.png", p_mig_sex_w, width = 7, height = 5, dpi = 600)

# --- Table

mig_table_sex_w <- migration_effects_sex %>%

mutate(

`Odds Ratio` = sprintf("%.2f", OR),

`95% CI` = paste0("[", sprintf("%.2f", CI_low), ", ", sprintf("%.2f", CI_high), "]"),

`p-value` = ifelse(p.value < 0.001, "<0.001", sprintf("%.3f", p.value))

) %>%

select(

Sex,

`Migration background` = term,

`Odds Ratio`, `95% CI`, `p-value`

)

gt_mig_sex_w <- gt(mig_table_sex_w, groupname_col = "Sex") %>%

tab_header(

title = "Effect of migration background on current smoking, stratified by sex",

subtitle = "Reference within each sex group: No migration background"

) %>%

cols_label(

`Migration background` = "Category"

)

gt_mig_sex_w

gtsave(gt_mig_sex_w, "stratified_migration_by_sex_weighted.docx")

# --- Forest-plot-ready dataset with reference rows

migration_effects_sex_fp <- migration_effects_sex %>%

mutate(

term = str_replace_all(term, "`", ""),

term = str_replace(term, "^statmigr", ""),

term = str_squish(term),

term = fct_relevel(term, "1st generation", "2nd or higher generation"),

reference = FALSE

)

ref_rows <- tibble(

Sex = c("Male","Female"),

term = factor("No migration background",

levels = c("No migration background","1st generation","2nd or higher generation")),

OR = 1,

CI_low = NA_real_,

CI_high = NA_real_,

p.value = NA_real_,

reference = TRUE

)

plot_df_sex <- migration_effects_sex_fp %>%

mutate(term = fct_expand(term, "No migration background")) %>%

bind_rows(ref_rows) %>%

group_by(Sex) %>%

mutate(term = fct_relevel(term, "No migration background", "1st generation", "2nd or higher generation")) %>%

ungroup()

rng <- plot_df_sex %>% filter(!reference, is.finite(CI_low), is.finite(CI_high))

xmin <- if (nrow(rng)) min(rng$CI_low) else 0.5

xmax <- if (nrow(rng)) max(rng$CI_high) else 2

xmin <- min(xmin, 0.5)

xmax <- max(xmax * 1.1, 1.5)

breaks_use <- c(0.25, 0.5, 1, 1.5, 2, 3, 5, 10)

breaks_use <- breaks_use[breaks_use >= xmin & breaks_use <= xmax]

p_fp_sex_w <- ggplot(plot_df_sex, aes(x = OR, y = term, shape = reference)) +

geom_vline(xintercept = 1, linetype = "dashed") +

geom_errorbarh(aes(xmin = CI_low, xmax = CI_high),

height = 0.18, linewidth = 0.5, na.rm = TRUE) +

geom_point(size = 2.6, stroke = 0.8, na.rm = TRUE) +

scale_shape_manual(values = c(`FALSE` = 16, `TRUE` = 4),

labels = c("Estimate","Reference")) +

scale_x_log10(breaks = breaks_use, limits = c(xmin, xmax)) +

facet_grid(rows = vars(Sex), scales = "free_y", space = "free_y",

switch = "y", as.table = FALSE) +

labs(

title = "Migration background and current smoking, stratified by sex",

subtitle = "Reference within each sex group: No migration background",

x = "Adjusted Odds Ratio (log scale)",

y = NULL,

shape = NULL

) +

theme_minimal(base_size = 11) +

theme(

legend.position = "right",

axis.text.y = element_text(size = 10),

strip.placement = "outside",

strip.text.y.left = element_text(angle = 0, hjust = 1, face = "bold"),

plot.title = element_text(face = "bold", hjust = 0.5),

panel.grid.minor = element_blank()

)

print(p_fp_sex_w)

ggsave("stratified_migration_sex_forest_weighted.png", p_fp_sex_w, width = 6.5, height = 5.5, dpi = 600)

ggsave("stratified_migration_sex_forest_weighted.pdf", p_fp_sex_w, width = 6.5, height = 5.5)

# --- Summary

sex_summary_w <- bind_rows(

tibble(

Sex = "Male",

N = fit_male$N,

Events = fit_male$Events,

Non_events = fit_male$Non_events,

Kept_covariates = paste(fit_male$Kept_covariates, collapse = ", ")

),

tibble(

Sex = "Female",

N = fit_female$N,

Events = fit_female$Events,

Non_events = fit_female$Non_events,

Kept_covariates = paste(fit_female$Kept_covariates, collapse = ", ")

)

)

print(sex_summary_w)

Stratified analysis 2: age

age_covars <- c(

"sex","statmigr","maritalstatus_recoded","AUSBILD3",

"erwerb","stala","sprache","alcohol_use","drug_use"

)

fit_one_age_stratum_w <- function(age_label, data, covars) {

ds <- data %>%

filter(alter7 == age_label) %>%

droplevels()

cov_keep <- drop_single_level_covars(ds, covars)

needed_vars <- c("smoker_binary_num", cov_keep, "wght")

ds <- ds %>%

filter(complete.cases(across(all_of(needed_vars))))

design_sub <- svydesign(

ids = ~1,

weights = ~wght,

data = ds

)

form <- reformulate(cov_keep, response = "smoker_binary_num")

fit <- svyglm(

form,

design = design_sub,

family = quasibinomial()

)

eff <- tidy_migration_weighted(fit, age_label, "Age")

list(

effects = eff,

N = nrow(ds),

Events = sum(ds$smoker_binary_num == 1, na.rm = TRUE),

Non_events = sum(ds$smoker_binary_num == 0, na.rm = TRUE),

model = fit,

Kept_covariates = cov_keep

)

}

age_levels <- levels(merged_data$alter7)

fits_age_w <- map(age_levels, ~ fit_one_age_stratum_w(.x, merged_data, age_covars))

migration_age_effects_w <- map_dfr(fits_age_w, "effects") %>%

mutate(

term = factor(term, levels = c("1st generation","2nd or higher generation")),

Age = factor(Age, levels = c("15–24","25–34","35–44","45–54","55–64","65–74","75+"), ordered = TRUE)

) %>%

arrange(Age, term)

age_N_summary_w <- tibble(

Age = age_levels,

N = map_int(fits_age_w, "N"),

Events = map_int(fits_age_w, "Events"),

Non_events = map_int(fits_age_w, "Non_events"),

Kept_covariates = map_chr(fits_age_w, ~ paste(.x$Kept_covariates, collapse = ", "))

) %>%

mutate(

Age = factor(Age, levels = c("15–24","25–34","35–44","45–54","55–64","65–74","75+"), ordered = TRUE)

) %>%

arrange(Age)

print(age_N_summary_w)

ref_rows <- tibble(

Age = factor(age_levels, levels = c("15–24","25–34","35–44","45–54","55–64","65–74","75+"), ordered = TRUE),

term = factor("No migration background",

levels = c("No migration background","1st generation","2nd or higher generation")),

OR = 1,

CI_low = NA_real_,

CI_high = NA_real_,

p.value = NA_real_,

reference = TRUE

)

plot_df_age_w <- migration_age_effects_w %>%

mutate(reference = FALSE,

term = fct_expand(term, "No migration background")) %>%

bind_rows(ref_rows) %>%

group_by(Age) %>%

mutate(term = fct_relevel(term, "No migration background", "1st generation", "2nd or higher generation")) %>%

ungroup() %>%

mutate(

Age = factor(Age,

levels = rev(c("15–24","25–34","35–44","45–54","55–64","65–74","75+")),

ordered = TRUE)

)

rng <- plot_df_age_w %>% filter(!reference, is.finite(CI_low), is.finite(CI_high))

xmin <- if (nrow(rng)) min(rng$CI_low) else 0.5

xmax <- if (nrow(rng)) max(rng$CI_high) else 2

xmin <- min(xmin, 0.5)

xmax <- max(xmax * 1.1, 1.5)

breaks_use <- c(0.25, 0.5, 1, 1.5, 2, 3, 5, 10, 20)

breaks_use <- breaks_use[breaks_use >= xmin & breaks_use <= xmax]

p_age_w <- ggplot(plot_df_age_w, aes(x = OR, y = term, shape = reference)) +

geom_vline(xintercept = 1, linetype = "dashed") +

geom_errorbarh(aes(xmin = CI_low, xmax = CI_high),

height = 0.2, linewidth = 0.5, na.rm = TRUE) +

geom_point(size = 2.6, stroke = 0.8, na.rm = TRUE) +

scale_shape_manual(values = c(`FALSE` = 16, `TRUE` = 4),

labels = c("Estimate","Reference")) +

scale_x_log10(breaks = breaks_use, limits = c(xmin, xmax)) +

facet_grid(rows = vars(Age),

scales = "free_y", space = "free_y",

switch = "y", as.table = FALSE) +

labs(

title = "Effect of migration background on current smoking, stratified by age group",

subtitle = "Reference within each age group: No migration background",

x = "Adjusted Odds Ratio (log scale)",

y = NULL,

shape = NULL

) +

theme_minimal(base_size = 11) +

theme(

legend.position = "right",

axis.text.y = element_text(size = 10),

strip.placement = "outside",

strip.text.y.left = element_text(angle = 0, hjust = 1, face = "bold"),

plot.title = element_text(face = "bold", hjust = 0.5),

panel.grid.minor = element_blank()

)

print(p_age_w)

ggsave("stratified_migration_by_age_weighted.png", p_age_w, width = 8, height = 9, dpi = 600)

ggsave("stratified_migration_by_age_weighted.pdf", p_age_w, width = 8, height = 9)

mig_age_table_w <- migration_age_effects_w %>%

mutate(

`Odds Ratio` = sprintf("%.2f", OR),

`95% CI` = paste0("[", sprintf("%.2f", CI_low), ", ", sprintf("%.2f", CI_high), "]"),

`p-value` = ifelse(p.value < 0.001, "<0.001", sprintf("%.3f", p.value))

) %>%

arrange(Age) %>%

select(

Age,

`Migration background` = term,

`Odds Ratio`, `95% CI`, `p-value`

)

gt_age_w <- gt(mig_age_table_w, groupname_col = "Age") %>%

tab_header(

title = "Effect of migration background on current smoking, stratified by age group",

subtitle = "Reference within each age group: No migration background"

)

gt_age_w

gtsave(gt_age_w, "stratified_migration_by_age_weighted.docx")

Stratified analysis 3: education

# EDUCATION

# ==== Education-stratified effects of migration background on current smoking ====

edu_covars <- c(

"sex","statmigr","alter7","maritalstatus_recoded",

"erwerb","stala","sprache","alcohol_use","drug_use"

)

fit_one_edu_stratum_w <- function(edu_label, data, covars) {

ds <- data %>%

filter(AUSBILD3 == edu_label) %>%

droplevels()

cov_keep <- drop_single_level_covars(ds, covars)

needed_vars <- c("smoker_binary_num", cov_keep, "wght")

ds <- ds %>%

filter(complete.cases(across(all_of(needed_vars))))

design_sub <- svydesign(

ids = ~1,

weights = ~wght,

data = ds

)

form <- reformulate(cov_keep, response = "smoker_binary_num")

fit <- svyglm(

form,

design = design_sub,

family = quasibinomial()

)

eff <- tidy_migration_weighted(fit, edu_label, "Education")

list(

effects = eff,

N = nrow(ds),

Events = sum(ds$smoker_binary_num == 1, na.rm = TRUE),

Non_events = sum(ds$smoker_binary_num == 0, na.rm = TRUE),

model = fit,

Kept_covariates = cov_keep

)

}

edu_levels <- levels(merged_data$AUSBILD3)

fits_edu_w <- map(edu_levels, ~ fit_one_edu_stratum_w(.x, merged_data, edu_covars))

migration_edu_effects_w <- map_dfr(fits_edu_w, "effects") %>%

mutate(

term = factor(term, levels = c("1st generation","2nd or higher generation")),

Education = factor(Education,

levels = c("Compulsory school or less","Secondary","Tertiary"),

ordered = TRUE)

) %>%

arrange(Education, term)

edu_N_summary_w <- tibble(

Education = edu_levels,

N = map_int(fits_edu_w, "N"),

Events = map_int(fits_edu_w, "Events"),

Non_events = map_int(fits_edu_w, "Non_events"),

Kept_covariates = map_chr(fits_edu_w, ~ paste(.x$Kept_covariates, collapse = ", "))

) %>%

mutate(

Education = factor(Education,

levels = c("Compulsory school or less","Secondary","Tertiary"),

ordered = TRUE)

) %>%

arrange(Education)

print(edu_N_summary_w)

ref_rows <- tibble(

Education = factor(edu_levels,

levels = c("Compulsory school or less","Secondary","Tertiary"),

ordered = TRUE),

term = factor("No migration background",

levels = c("No migration background","1st generation","2nd or higher generation")),

OR = 1,

CI_low = NA_real_,

CI_high = NA_real_,

p.value = NA_real_,

reference = TRUE

)

plot_df_edu_w <- migration_edu_effects_w %>%

mutate(reference = FALSE,

term = fct_expand(term, "No migration background")) %>%

bind_rows(ref_rows) %>%

group_by(Education) %>%

mutate(term = fct_relevel(term, "No migration background", "1st generation", "2nd or higher generation")) %>%

ungroup() %>%

mutate(

Education = factor(Education,

levels = c("Tertiary","Secondary","Compulsory school or less"),

ordered = TRUE)

)

rng <- plot_df_edu_w %>% filter(!reference, is.finite(CI_low), is.finite(CI_high))

xmin <- if (nrow(rng)) min(rng$CI_low) else 0.5

xmax <- if (nrow(rng)) max(rng$CI_high) else 2

xmin <- min(xmin, 0.5)

xmax <- max(xmax * 1.1, 1.5)

breaks_use <- c(0.25, 0.5, 1, 1.5, 2, 3, 5, 10, 20)

breaks_use <- breaks_use[breaks_use >= xmin & breaks_use <= xmax]

# --- Forest plot (faceted by Education) --------------------------------------

p_edu_w <- ggplot(plot_df_edu_w, aes(x = OR, y = term, shape = reference)) +

geom_vline(xintercept = 1, linetype = "dashed") +

geom_errorbarh(aes(xmin = CI_low, xmax = CI_high),

position = position_dodge(width = 0.6),

height = 0.2, linewidth = 0.5, na.rm = TRUE) +

geom_point(position = position_dodge(width = 0.6),

size = 2.6, stroke = 0.8, na.rm = TRUE) +

scale_shape_manual(values = c(`FALSE` = 16, `TRUE` = 4),

labels = c("Estimate","Reference")) +

scale_x_log10(breaks = breaks_use) +

coord_cartesian(xlim = c(xmin, xmax)) +

facet_grid(rows = vars(Education),

scales = "free_y", space = "free_y",

switch = "y", as.table = TRUE) +

labs(

title = "Migration background and current smoking, stratified by education",

subtitle = "Reference within each education group: No migration background",

x = "Adjusted Odds Ratio (log scale)",

y = NULL,

shape = NULL

) +

theme_minimal(base_size = 11) +

theme(

legend.position = "right",

axis.text.y = element_text(size = 10),

strip.placement = "outside",

strip.text.y.left = element_text(angle = 0, hjust = 1, face = "bold"),

plot.title = element_text(face = "bold", hjust = 0.5, margin = margin(b = 6)),

plot.subtitle = element_text(hjust = 0.5),

plot.title.position = "plot",

plot.margin = margin(t = 10, r = 30, b = 10, l = 10),

panel.grid.minor = element_blank()

)

print(p_edu_w)

ggsave("stratified_migration_by_education_weighted.png", p_edu_w, width = 7.5, height = 7.5, dpi = 600)

ggsave("stratified_migration_by_education_weighted.pdf", p_edu_w, width = 7.5, height = 7.5)

mig_edu_table_w <- migration_edu_effects_w %>%

mutate(

`Odds Ratio` = sprintf("%.2f", OR),

`95% CI` = paste0("[", sprintf("%.2f", CI_low), ", ", sprintf("%.2f", CI_high), "]"),

`p-value` = ifelse(p.value < 0.001, "<0.001", sprintf("%.3f", p.value))

) %>%

arrange(Education) %>%

select(

Education,

`Migration background` = term,

`Odds Ratio`, `95% CI`, `p-value`

)

gt_edu_w <- gt(mig_edu_table_w, groupname_col = "Education") %>%

tab_header(

title = "Effect of migration background on current smoking, stratified by education",

subtitle = "Reference within each education group: No migration background"

)

gt_edu_w

gtsave(gt_edu_w, "stratified_migration_by_education_weighted.docx")

## ------------------------------------------------------------

# TOTAL N SUMMARY

## ------------------------------------------------------------

cat("\n=== TOTAL N (weighted complete-case analytic subsets; raw counts shown) ===\n")

N_main_w <- nrow(analysis_data_main)

cat("Main weighted model: N =", N_main_w, "\n")

N_sex_w <- tibble(

Sex = c("Male","Female"),

N = c(fit_male$N, fit_female$N)

) %>%

mutate(Total = sum(N))

print(N_sex_w)

N_age_w <- tibble(

Age = age_levels,

N = map_int(fits_age_w, "N")

) %>%

mutate(Total = sum(N))

print(N_age_w)

N_edu_w <- tibble(

Education = edu_levels,

N = map_int(fits_edu_w, "N")

) %>%

mutate(Total = sum(N))

print(N_edu_w)

cat("\n=== Consistency check ===\n")

cat("Sum of sex-strata N equals main N? ", sum(N_sex_w$N) == N_main_w, "\n")

cat("Sum of age-strata N equals main N? ", sum(N_age_w$N) == N_main_w, "\n")

cat("Sum of education-strata N equals main N? ", sum(N_edu_w$N) == N_main_w, "\n")
